# Supplementary material for: A prospective study of consecutive emergency medical admissions to compare a novel automated computer-aided mortality risk score and clinical judgement of patient mortality risk
Source: BMJ Open. 2019 Jun 19;9(6):e027741. doi: 10.1136/bmjopen-2018-027741 (PMC6589037; doi:10.1136/bmjopen-2018-027741)
Supplement: Author's manuscript [file bmjopen-2018-027741.draft_revisions.pdf]

BMJ Open is committed to open peer review. As part of this commitment we make the peer review history of every article we publish publicly available.

When an article is published we post the peer reviewers' comments and the authors' responses online. We also post the versions of the paper that were used during peer review. These are the versions that the peer review comments apply to.

The versions of the paper that follow are the versions that were submitted during the peer review process. They are not the versions of record or the final published versions. They should not be cited or distributed as the published version of this manuscript.

BMJ Open is an open access journal and the full, final, typeset and author-corrected version of record of the manuscript is available on our site with no access controls, subscription charges or pay-per-view fees (http://bmjopen.bmj.com).

If you have any questions on BMJ Open's open peer review process please email info.bmjopen@bmj.com

BMJ Open

A novel automated computer aided risk of mortality score compares favourably with medical judgement in predicting a patient's risk of mortality following emergency medical admission

Journal:	*BMJ Open*
Manuscript ID	bmjopen-2018-027741
Article Type:	Research
Date Submitted by the Author:	06-Nov-2018
Complete List of Authors:	Faisal, Muhammad; University of Bradford,<br>Khatoon, Binish; University of Bradford Faculty of Health Studies<br>Scally, Andy; University College Cork National University of Ireland, School of Clinical Therapies<br>Richardson, Donald; York Teaching Hospital NHS Foundation Trust, Renal Unit<br>Irwin, Sally; York Teaching Hospital NHS Foundation Trust<br>Davidson, Rachel; York Teaching Hospital NHS Foundation Trust<br>Heseltine, David; York Teaching Hospital NHS Foundation Trust<br>Corlett, Alison; York Teaching Hospital NHS Foundation Trust<br>Ali, Javed; York Teaching Hospital NHS Foundation Trust<br>Hampson, Rebecca; York Teaching Hospital NHS Foundation Trust<br>Kesavan, Sandeep; York Teaching Hospital NHS Foundation Trust<br>McGonigal, Gerry; York Teaching Hospital NHS Foundation Trust<br>Goodman, Karen; York Teaching Hospital NHS Foundation Trust<br>Harkness, Michael; York Teaching Hospital NHS Foundation Trust<br>Mohammed, Mohammed ; University of Bradford Faculty of Health Studies; Bradford Institute for Health Research
Keywords:	computer aided-risk score, medical judgement, mortality, emergency medical admission

SCHOLARONE™
Manuscripts

A novel automated computer aided risk of mortality score compares favourably with medical judgement in predicting a patient's risk of mortality following emergency medical admission

Authors

Muhammad Faisal, PhD
Senior Research Fellow in Medical Statistics
Faculty of Health Studies, University of Bradford, Bradford, UK
Bradford Institute for Health Research
E-mail: M.Faisal1@bradford.ac.uk

Binish Khatoon, PhD
Research Fellow
Faculty of Health Studies, University of Bradford, Bradford, UK
Bradford Institute for Health Research
E-mail: B.Khatoon@bradford.ac.uk

Andy Scally, MSc
Senior Lecturer
School of Clinical Therapies
University College Cork, Ireland
E-mail: andrew.scally@ucc.ie

Donald Richardson, FRCP
Consultant Renal Physician
Department of Renal Medicine, York Teaching Hospital NHS Foundation Trust Hospital
E-mail: drichardson@doctors.org.uk

Sally Irwin FRCP
Consultant Geriatrician,
Department of Elderly Medicine,
York Teaching Hospital NHS Foundation Trust Hospital
Email:sally.irwin@york.nhs.uk

Rachel Davidson MRCP
Consultant Geriatrician,
Department of Elderly Medicine,
York Teaching Hospital NHS Foundation Trust Hospital
Email:Rachel.davidson@york.nhs.uk

David Heseltine FRCP
Consultant Geriatrician,
Department of Elderly Medicine,
York Teaching Hospital NHS Foundation Trust Hospital
Email:david.heseltine@york.nhs.uk

Alison Corlett FRCP
Consultant Geriatrician,

For peer review only

Department of Elderly Medicine,
York Teaching Hospital NHS Foundation Trust Hospital
Email: Alison.j.corlett@york.nhs.uk

Javed Ali MRCP (Ireland)
Consultant Geriatrician,
Department of Elderly Medicine,
York Teaching Hospital NHS Foundation Trust Hospital
Email:Javed.ali@york.nhs.uk

Rebecca Hampson MRCP
Consultant Geriatrician,
Department of Elderly Medicine,
York Teaching Hospital NHS Foundation Trust Hospital
Email: Rebecca.hampson@york.nhs.uk

Sandeep Kesavan FRCP (Edin)
Consultant Geriatrician,
Department of Elderly Medicine,
York Teaching Hospital NHS Foundation Trust Hospital
Email: Sandeep.kesavan@york.nhs.uk

Gerry McGonigal MD
Consultant Geriatrician,
Department of Elderly Medicine,
York Teaching Hospital NHS Foundation Trust Hospital
Email: Gerard.mcgonigal@york.nhs.uk

Karen Goodman FRCP (Edin)
Consultant Geriatrician,
Department of Elderly Medicine,
York Teaching Hospital NHS Foundation Trust Hospital
Email:Karen.goodman@york.nhs.uk

Michael Harkness FRCP
Consultant Geriatrician,
Department of Elderly Medicine,
York Teaching Hospital NHS Foundation Trust Hospital
Email: Michael.harkness@york.nhs.uk

Mohammed A Mohammed, PhD
Professor of Healthcare Quality & Effectiveness
Faculty of Health Studies, University of Bradford, Bradford, UK
Deputy Director of the Bradford Institute for Health Research
Academic Director to the Yorkshire & Humberside Academic Health Sciences Network
E-mail: M.A.Mohammed5@Bradford.ac.uk

Correspondence to Mohammed A Mohammed

Word-count: 2491

For peer review only

Abstract

Objective: To compare the performance of an automated validated computer aided risk of mortality score (CARM) versus medical judgment in predicting the risk of in-hospital mortality for patients following emergency medical admission.

Method: Consecutive emergency admissions to an elderly care medical admissions ward in one hospital were assigned a risk of death at the first post take ward round by consultant staff over a two-week period. The same admissions were subsequently assigned a risk of death using the CARM score, based on age, sex, vital signs and blood test results. The performance of the CARM versus consultant medical judgement was compared using the area under the ROC curve (c-statistic) and the positive predictive value (PPV).

Results: The in-hospital mortality was 33.1% (121/366). The c-statistic for CARM was 0.75 (95% CI 0.70 to 0.80) (CARM) versus 0.72 (95% CI 0.67 to 0.77) for medical judgements. The PPV at a 5% and 10% risk threshold was higher for CARM (47.0%, 61.9%) compared to medical judgement (43.9%, 51.5%).

Conclusion: CARM compares favourably with medical judgements in routine clinical care. CARM appears to have a promising role in supporting medical judgements in determining the patient's risk of death in hospital. Further evaluation of CARM in routine practice is required.

Keywords: computer aided-risk score; medical judgement; mortality; emergency medical admission

For peer review only

Article Summary

- This study compares a novel computer-aided risk of mortality (CARM) score versus medical judgment in predicting the risk of in-hospital mortality.
- Consecutive emergency admissions to an elderly care medical admissions ward in one hospital were assigned a risk of death at the first post take ward round by consultant staff.
- We then compared the performance of the CARM with consultant medical judgement score using the area under the ROC curve (c-statistic) and the positive predictive value (PPV)
- About 12% of admissions do not have both NEWS and blood test results and so CARM is not applicable to these admissions.

For peer review only

Introduction

Over the past three decades, numerous scoring systems have been developed to estimate the risk of mortality in patients admitted to hospital. Two of the most frequently used scores are Acute Physiology and Chronic Health Evaluation (APACHE2) [1], and Simplified Acute Physiology Score (SAPS) [2]. Nonetheless, despite the preponderance of scoring systems, few studies [3–5] have assessed the accuracy of risk equations versus medical judgments in routine clinical settings. This is important because if the risk score is found not to perform well when compared to medical judgements, this would call into question the incremental benefit of using the score in routine clinical practice despite the pedigree of the risk score.

The National Early Warning Score (NEWS) is based on the patients' vital signs and in widespread use across hospitals in the English National Health Service (NHS) and there has been interest in the utilisation of that score to guide escalation of care in adult in-patient settings. The score is not presented as a mortality risk but as a numeric score (0 to 19 maximum) with higher scores reflecting more sever sickness. The scores are linked to local hospital escalations of care policies. The mortality risk across NEWS has previously been published [6] but this specific association may not be widely recognised by frontline clinical staff.

We recently developed a validated computer aided risk of in-hospital mortality (CARM) score, which combines age, sex, vital signs (based on NEWS) and blood test results for emergency medical admissions [7]. Since all the data items used in CARM are routinely collected as part of the process of care there is no additional data collection burden on clinical staff and as soon as the data items are electronically recorded the CARM score is automatically computed. As part of the evaluation of CARM we set out to compare the performance of CARM versus medical judgements in estimating the risk of in-hospital mortality in consecutive emergency admissions to an elderly care ward in one hospital over a two-week period.

For peer review only

Methods

Setting & data

Our cohort of elderly medical admissions is from York Hospital (managed by York Teaching Hospitals NHS Foundation Trust) which has approximately 700 beds. It has been exclusively using electronic NEWS scoring since at least 2013 as part of their in-house electronic patient record systems. Consecutive admissions to an elderly care medical admissions ward in this hospital were assigned a risk of death at the first post take ward round by consultant medical staff over a two-week period (February 05, 2017 to February 20, 2017). The medical staff did not have access to the CARM score during the data collection exercise. The same admissions, providing they had sufficient data to derive a CARM score, were subsequently assigned a risk of death using the CARM score, based on their age, sex, vital signs (based on NEWS) and blood test results [7]. For each admission, we obtained the patient's age, sex (male/female), admission and discharge date and time, AKI score, electronic National Early Warning Score (NEWS) (including its subcomponent vital signs data), and seven blood test results (albumin, creatinine, haemoglobin, potassium, sodium, urea, and white cell count). We excluded records where blood test results were not undertaken at all. However, we imputed population age and sex specific median albumin if missing because this is not routinely included in the list of routine blood tests at York Hospital.

Statistical Analysis

The performance of CARM versus medical judgement was assessed by comparing risk estimates using boxplots. The discrimination of CARM and medical judgments was quantified by the area under the Receiver-Operating Characteristic (ROC) curve [8]. The ROC curve is a plot of the sensitivity, (true positive rate), versus 1-specificity, (false positive rate). The area under the ROC curve is summarised by a c-statistic which is interpreted as the probability that a randomly chosen deceased patient has a higher risk of death than a randomly chosen non-deceased patient. A c-statistic or AUC of 0.5 is no better than tossing a coin, whilst a perfect model has a c-statistic of 1. The higher the c-statistic, the

For peer review only

better the discrimination. In general, values less than 0.7 are considered to show poor discrimination, values of 0.7–0.8 can be described as reasonable, and values above 0.8 suggest good discrimination [9]. We compared AUC for CARM and medical judgement using the DeLong's test [10].

We further determined the sensitivity, specificity, positive and negative predictive values, positive and negative likelihood ratios for CARM and compared this with medical judgement scores using probability thresholds from a NEWS only model for NEWS scores from 1 to 5. The cut-off of NEWS at 5 is the recommended threshold for escalation of care [11,12]. All analyses were undertaken in STATA [13] and R [14] using *rms* [15] and *pROC* [16] packages.

Ethical approval

This study received ethical approval from The Yorkshire & Humberside Leeds West Research Ethics Committee on 17 September 2015 (ref. 173753) with NHS management permissions received January 2016.

Patient and Public Involvement

A workshop with a patient and service user group, linked to the University of Bradford, was involved at the start of this project to co-design the agenda for the patient and staff focus groups which were subsequently held at each hospital site. Patients were invited to attend the patient focus group through existing patient and public involvement groups. The criteria used for recruitment to these focus group was any member of the public who had been a patient or carer in the last five years. The patient and public voice continued to be included throughout the project with three patient representatives invited to sit on the project steering group. Participants will be informed of the results of this study through the patient and public involvement leads at each hospital site and the project team have met with the Bradford Patient and service user group to discuss the results.

For peer review only

Data Sharing Statement

Our data sharing agreement with the York hospital does not permit us to share this data with other parties. Nonetheless if anyone is interested in the data, then they should contact the R&D offices at York hospital in the first instance.

Results

Cohort description

We considered 414 elderly medical admissions in York hospital. Of these 48 (11.6%) were not eligible for comparison because no or incomplete blood test results were recorded (Table 1).

Characteristic	N (%)	Died (%)
Total emergency medical admissions	414	131 (31.6)
Total excluded: No or incomplete blood test results recorded (%) [excepting Albumin]	48 (11.6)	10 (20.8)
Total included (%)	366 (88.4)	121 (33.1)

Table 1 Number and mortality of elderly medical admissions included/excluded

The in-hospital mortality was 31.7% (121/366). The age, sex, NEWS and blood test results profile is shown in Table 2. Compared with patients discharged alive, the deceased patients were aged older, with lower albumin, haemoglobin and sodium values, and higher creatinine, potassium, white cell count and urea values. NEWS was higher in deceased patients compared with patients discharged alive, as were respiratory rate and pulse rate values. The temperature, blood pressure and oxygen saturation values were lower in deceased patients.

Characteristic	Discharged alive	Discharged deceased
N	245	121
Male (%)	109 (44.5)	65 (53.7)
Mean CARM Score (SD)	0.07 (0.07)	0.17 (0.16)
Mean Medical Judgement Risk Score (SD)	0.13 (0.14)	0.27 (0.26)
Mean NEWS (SD)	2 (2.1)	3.3 (3.2)
Alertness		
Alert (%)	244 (99.6)	114 (94.2)
Pain (%)	0 (0.0)	3 (2.5)

For peer review only

Voice (%)	1 (0.4)	4 (3.3)
Unconscious (%)	0 (0.0)	0 (0.0)
AKI Score		
0 (%)	237 (96.7)	113 (93.4)
1 (%)	5 (2.0)	5 (4.1)
2 (%)	2 (0.8)	2 (1.7)
3 (%)	1 (0.4)	1 (0.8)
Oxygen supplementation (%)	45 (18.4)	40 (33.1)
Mean Age [years] (SD)	84.2 (5.3)	86.9 (6.5)
Mean Albumin [g/L] (SD)	36.6 (4.0)	34.1 (5.4)
Mean Creatinine [umol/L] (SD)	105.3 (60.7)	120.2 (76.4)
Mean Haemoglobin [g/l] (SD)	122.1 (20.1)	117.7 (18.1)
Mean Potassium [mmol/L] (SD)	4.3 (0.5)	4.4 (0.6)
Mean Sodium [mmol/L] (SD)	135.9 (4.4)	135.5 (5.8)
Mean White cell count [10^9 cells/L] (SD)	10.4 (6.6)	12 (13.1)
Mean Urea [mmol/L] (SD)	9.3 (5.5)	12.5 (9)
Mean Respiratory rate [breaths per minute] (SD)	18.4 (2.9)	19.2 (4.5)
Mean Temperature [^{o}C] (SD)	36.5 (0.7)	36.4 (0.8)
Mean Systolic pressure [mmHg] (SD)	134 (24.5)	122.5 (21.8)
Mean Diastolic pressure [mmHg] (SD)	70.7 (13.9)	67.6 (12.1)
Mean Pulse rate [beats per minute] (SD)	78.5 (16.5)	81.5 (18.6)
Mean % Oxygen saturation (SD)	96.2 (1.9)	95.5 (3.1)

Table 2 Characteristics of elderly medical admissions.

For peer review only

Comparison of CARM versus Medical Judgement

The boxplots in Figure 1 show that the (estimated) risk of in-hospital mortality using CARM versus medical judgments for patients discharge alive and deceased. The predicted risk is systematically lower using CARM than for medical judgement for both patients who were discharged alive and deceased. The mean estimated risk of in-hospital mortality for patients discharged alive was lower with CARM (0.07 SD=0.07) versus medical judgements (0.13 SD=0.14). Likewise for decreased patients, the risk estimates from CARM (0.17 SD=0.16) were lower than estimates from medical judgements (0.27 SD=0.26). Figure 2 shows the ROC curve. The area under the ROC curve (c-statistic), was higher for CARM 0.75 (95% CI 0.70 to 0.80) than for medical judgement 0.72 (95% CI 0.67 to 0.77) and were not statistically significant (p-value = 0.34).

Table 3 shows the sensitivity, specificity, positive predictive value and negative predictive value for a selected range NEWS values. NEWS at 5 (the recommended escalation threshold), which corresponds to a 10% risk of in-hospital mortality, medical judgement had a higher sensitivity (57.9% vs 53.7%), lower specificity (73.1% vs 83.7%), lower PPVs (51.5% vs 61.9%) and lower positive likelihood ratios (2.1 vs 3.3).

For peer review only

NEWS	Predicted risk at NEWS thresholds	Medical Judgement							CARM						
		N	Sensitivity%	Specificity%	PPV	NPV	LR+	LR-	N	Sensitivity%	Specificity%	PPV	NPV	LR+	LR-
1	0.03	331	98.3 (94.2 to 99.8)	13.5 (9.5 to 18.4)	36 (30.8 to 41.4)	94.3 (80.8 to 99.3)	1.1 (1.1 to 1.2)	0.1 (0 to 0.5)	289	91.7 (85.3 to 96)	27.3 (21.9 to 33.4)	38.4 (32.8 to 44.3)	87 (77.4 to 93.6)	1.3 (1.1 to 1.4)	0.3 (0.2 to 0.6)
2	0.04	329	98.3 (94.2 to 99.8)	14.3 (10.2 to 19.3)	36.2 (31 to 41.6)	94.6 (81.8 to 99.3)	1.1 (1.1 to 1.2)	0.1 (0 to 0.5)	245	84.3 (76.6 to 90.3)	41.6 (35.4 to 48.1)	41.6 (35.4 to 48.1)	84.3 (76.6 to 90.3)	1.4 (1.3 to 1.6)	0.4 (0.2 to 0.6)
3	0.05	228	82.6 (74.7 to 88.9)	47.8 (41.4 to 54.2)	43.9 (37.3 to 50.6)	84.8 (77.7 to 90.3)	1.6 (1.4 to 1.8)	0.4 (0.2 to 0.5)	200	77.7 (69.2 to 84.8)	56.7 (50.3 to 63)	47.0 (39.9 to 54.2)	83.7 (77.2 to 89)	1.8 (1.5 to 2.1)	0.4 (0.3 to 0.6)
4	0.08	224	81.8 (73.8 to 88.2)	49 (42.6 to 55.4)	44.2 (37.6 to 51)	84.5 (77.5 to 90)	1.6 (1.4 to 1.9)	0.4 (0.2 to 0.6)	149	62.8 (53.6 to 71.4)	70.2 (64.1 to 75.9)	51 (42.7 to 59.3)	79.3 (73.3 to 84.5)	2.1 (1.7 to 2.7)	0.5 (0.4 to 0.7)
5	0.10	136	57.9 (48.5 to 66.8)	73.1 (67 to 78.5)	51.5 (42.8 to 60.1)	77.8 (71.9 to 83)	2.1 (1.7 to 2.8)	0.6 (0.5 to 0.7)	105	53.7 (44.4 to 62.8)	83.7 (78.4 to 88.1)	61.9 (51.9 to 71.2)	78.5 (73.1 to 83.4)	3.3 (2.4 to 4.6)	0.6 (0.5 to 0.7)

Table 3 Performance of CARM versus medical judgement in predicting the risk in-hospital mortality at NEWS thresholds (1, 2, 3, 4, 5)

PPV=Positive Predictive Value; NPV= Negative Predictive Value; LR+=Positive Likelihood Ratio; LR-=Negative Likelihood Ratio

For peer review only

Discussion

In this study, we found the CARM compares favourably with medical judgements made by consultant medical staff in-predicting the risk of in-hospital mortality for emergency medical patients admitted to the elderly care ward. CARM has a comparable discrimination, and higher PPV and positive likelihood ratios. These findings are remarkable because, unlike medical judgements, CARM relies exclusively on routinely collected data based primarily on the patients' vital signs and blood test results without having any disease labels or clinical history. Nonetheless it is important to note that we have designed CARM to support the medical decision-making process, not replace it, without placing any additional data collection burden on staff. The CARM risk prediction can also be made available as soon as the physiological observations and blood test results are available and prior to the consultant review which may be of assistance to more junior staff. CARM was developed using all adult non-elective admissions to medicine and elderly in one trust and externally validated in other trust [7].

Our study has several limitations. This study provides a snapshot of the use of CARM in a hospital over a short period and the extent to which our findings generalise to patients over a longer time period and to other wards and hospitals merit further study. Although CARM is designed to be automated, we note that for 11.6% of patients were unable to derive the CARM score because of no or incomplete blood test results. The impact of this design feature of CARM in routine clinical practice remain to be seen. For example, there may be an increase in the use of blood test results in patients where blood test would not ordinarily be undertaken to simply provide a CARM score. Furthermore, how the systematically lower estimated risks from CARM actually interact with and modify medical judgments also merits further study.

The overall mortality was 5% in the study population in which the CARM risk predictor was developed. The overall mortality in this patient cohort is high and it is worth noting that patients had already been streamed (selected) as requiring in-patient admission as direct admission from GP or via the

For peer review only

emergency department. Thus the pre-test probability of mortality is different to original study population yet the CARM risk predictor still performs well in this population. Alternative pathways exist for specialty patients, frailty and ambulatory patients within the hospital involved in the study where the mean mortality for elderly care non-elective patients is 8%.

When comparing CARM with medical judgments, no significant differences in AUC were observed. Our findings are in line with other study, which also found no significant differences between ROC curves for APACHE2 and clinical staff [17]. However, a study reported that the clinical assessment had an overall accuracy of 95.2% versus 90.9% for APACHE2 [3]. Other studies have also failed to show an advantage for the APACHE2 model when compared to medical judgments by the clinicians [4,5,18]. Another study found that physicians were significantly better in predicting outcome in a medical intensive care unit than APACHE [19]. One study concluded that physicians' clinical judgment could differ from scoring systems enough to account for large differences in expected outcomes [18].

Although our results are promising, further more rigorous evaluation of CARM is required in real-time routine clinical practice over longer time scales and with a wider variety of patients and medical staff. The key outstanding question is to determine the extent to which medical decision making and the quality and safety of care are enhanced by the use of CARM.

The risk score is produced by an algorithm using variables that are already available to the clinician. These variables are however ‘processed’ and a risk score is synthetically created where the human brain is not capable of performing these calculations in real time. Clinicians predominantly use rule based decisions making, experiential decision making models and a combination of the two as they become more experienced/develop expertise. A risk prediction score is not there to replace the clinical skills and human interface that exists between patient and clinician, but it may be able to improve the situational awareness of the clinician, particularly those with less experience than the consultant. Further studies of the utilisation of risk scores in the clinical environment are required.

For peer review only

Conclusions

CARM compares favorably with consultant level medical judgements and in routine clinical care. CARM appears to have a promising role in supporting medical judgements in determining the patient's risk of death in hospital. Further evaluation of CARM in practice is required.

Funding

This research was supported by the Health Foundation. The Health Foundation is an independent charity working to improve the quality of health care in the UK.

This research was supported by the National Institute for Health Research (NIHR) Yorkshire and Humberside Patient Safety Translational Research Centre (NIHR YHPSTRC). The views expressed in this article are those of the author(s) and not necessarily those of the NHS, the NIHR, or the Department of Health and Social Care.

Contributorship

MAM & DR had the original idea for this work. MF and MAM undertook the statistical analyses. DR gave a clinical perspective. MF and BK wrote the first draft of this paper. All other authors contributed to data collection and all authors subsequently assisted in redrafting and have approved the final version. MAM will act as guarantor.

Competing Interests: The authors declare no conflicts of interest.

References

[1] Knaus WA, Draper EA, Wagner DP, Zimmerman JE. APACHE II: A severity of disease classification system. Crit Care Med 1985;13:818–29. doi:10.1097/00003246-198510000-00009.

[2] Le Gall JR, Loirat P, Alperovitch A, Glaser P, Granthil C, Mathieu D, et al. A simplified acute physiology score for ICU patients. Crit Care Med 1984;12:975–7.

[3] Meyer AA, Messick WJ, Young P, Baker CC, Fakhry S, Muakkassa F, et al. Prospective comparison of clinical judgment and APACHE II score in predicting the outcome in critically ill surgical patients. J Trauma 1992;32:744–7.

[4] McClish DK, Powell SH. How Well Can Physicians Estimate Mortality in a Medical Intensive Care Unit? Med Decis Mak 1989;9:125–32. doi:10.1177/0272989X8900900207.

[5] Christensen C, J Cottrell J, Murakami J, E Mackesy M, S Fetzer A, Elstein A. Forecasting Survival in the Medical Intensive Care Unit: A Comparison of Clinical Prognoses With Formal Estimates. vol. 32. 1993. doi:10.1055/s-0038-1634937.

[6] Royal College of Physicians. National Early Warning Score (NEWS): Standardising the assessment of acuteillness severity in the NHS - Report of a working party. 2012.

For peer review only

[7] Faisal M, Scally A, Jackson N, Richardson D, Beatson K, Howes R, et al. Development and validation of a novel computer-aided score to predict the risk of in-hospital mortality for acutely ill medical admissions using their first electronically recorded blood test results and vital signs. BMJ Open (in Press 2018.
[8] Steyerberg EW, Vickers AJ, Cook NR, Gerds T, Gonen M, Obuchowski N, et al. Assessing the performance of prediction models: a framework for traditional and novel measures. Epidemiology 2010;21:128–38. doi:10.1097/EDE.0b013e3181c30fb2.
[9] Hanley JA, McNeil BJ. The meaning and use of the area under a receiver operating characteristic (ROC) curve. Radiology 1982;143:29–36. doi:10.1148/radiology.143.1.7063747.
[10] DeLong ER, DeLong DM, Clarke-Pearson DL. Comparing the areas under two or more correlated receiver operating characteristic curves: a nonparametric approach. Biometrics 1988;44:837–45.
[11] Goulden R, Hoyle M-C, Monis J, Railton D, Riley V, Martin P, et al. qSOFA, SIRS and NEWS for predicting inhospital mortality and ICU admission in emergency admissions treated as sepsis. Emerg Med J 2018;35:345–9. doi:10.1136/emermed-2017-207120.
[12] NHS. Royal College of Physicians: NHS England approves use of National Early Warning Score (NEWS) 2 to improve detection of acutely ill patients. 2017.
[13] StatCorp. Stata: Release 14. Statistical Software. College Station, TX: StataCorp LP 2016.
[14] R Development Core Team. R: A language and environment for statistical computing. R Foundation for Statistical Computing http://www.r-project.org/ 2015.
[15] Harrell FE. rms: Regression Modeling Strategies http://cran.r-project.org/package=rms 2015.
[16] Robin X, Turck N, Hainard A, Tiberti N, Lisacek F, Sanchez JJ-CC, et al. pROC: an open-source package for R and S+ to analyze and compare ROC curves. BMC Bioinformatics 2011;12:77. doi:10.1186/1471-2105-12-77.
[17] Kruse JA, Thill-Baharozian MC, Carlson RW. Comparison of clinical assessment with APACHE II for predicting mortality risk in patients admitted to a medical intensive care unit. JAMA 1988;260:1739–42.
[18] L. PG, M. PC. Physician risk assessment and apache scores in cardiac care units. Clin Cardiol 2009;22:366–8. doi:10.1002/clc.4960220514.
[19] Brannen AL 2nd, Godfrey LJ, Goetter WE. Prediction of outcome from critical illness. A comparison of clinical judgment with a prediction rule. Arch Intern Med 1989;149:1083–6.

For peer review only

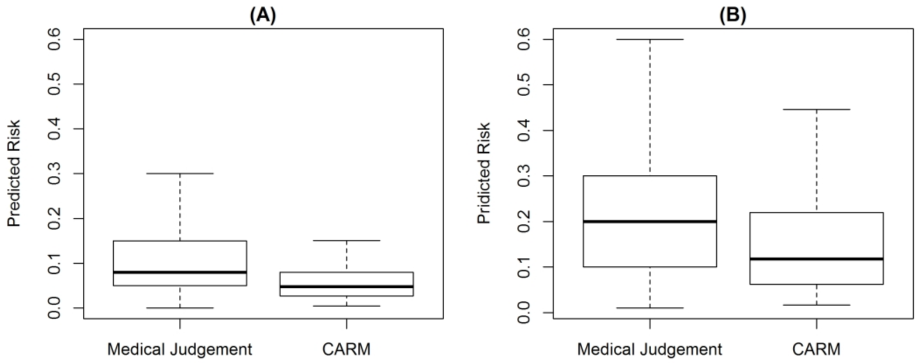

Boxplots for CARM versus medical judgement for patients who (A) discharged alive and (B) discharged deceased.

254x114mm (300 x 300 DPI)

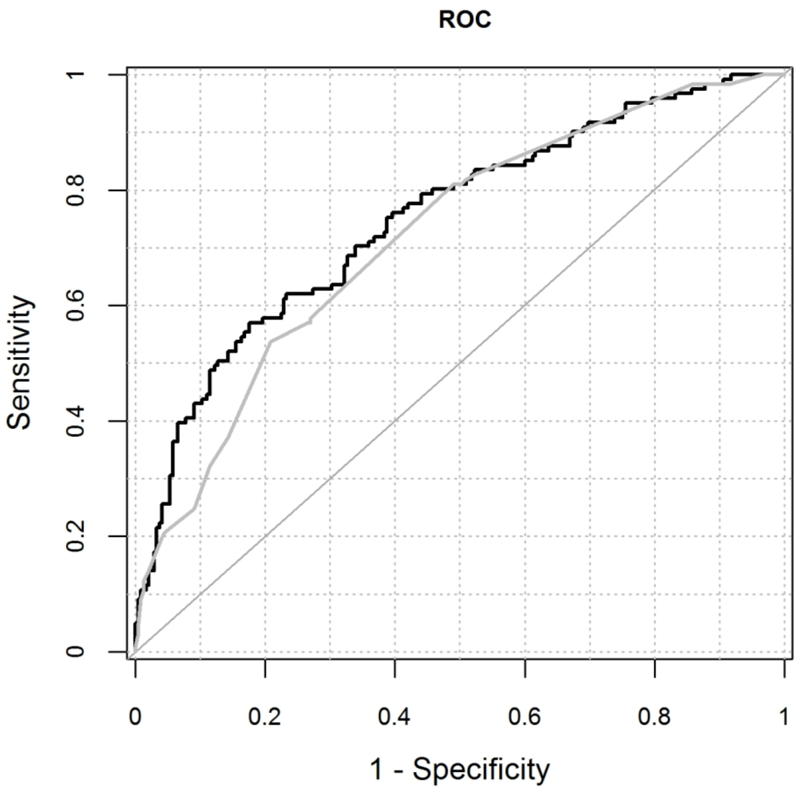

Receiver Operating Characteristic curve for CARM (c-statistic 0.75) and medical judgements (c- statistic 0.72)
Black line is for CARM and grey line is for medical judgement

139x127mm (300 x 300 DPI)

A novel automated computer aided risk of mortality score is comparable to medical judgement in predicting a patient's risk of mortality following emergency medical admission: A prospective study of consecutive patients.

Journal:	*BMJ Open*
Manuscript ID	bmjopen-2018-027741.R1
Article Type:	Research
Date Submitted by the Author:	08-Apr-2019

Complete List of Authors:	Faisal, Muhammad; University of Bradford,<br>Khatoon, Binish; University of Bradford Faculty of Health Studies<br>Scally, Andy; University College Cork National University of Ireland, School of Clinical Therapies<br>Richardson, Donald; York Teaching Hospital NHS Foundation Trust, Renal Unit<br>Irwin, Sally; York Teaching Hospital NHS Foundation Trust<br>Davidson, Rachel; York Teaching Hospital NHS Foundation Trust<br>Heseltine, David; York Teaching Hospital NHS Foundation Trust<br>Corlett, Alison; York Teaching Hospital NHS Foundation Trust<br>Ali, Javed; York Teaching Hospital NHS Foundation Trust<br>Hampson, Rebecca; York Teaching Hospital NHS Foundation Trust<br>Kesavan, Sandeep; York Teaching Hospital NHS Foundation Trust<br>McGonigal, Gerry; York Teaching Hospital NHS Foundation Trust<br>Goodman, Karen; York Teaching Hospital NHS Foundation Trust<br>Harkness, Michael; York Teaching Hospital NHS Foundation Trust<br>Mohammed, Mohammed ; University of Bradford Faculty of Health Studies; NHS Midlands and Lancashire Commissioning Support Unit

**Primary Subject Heading**:	Health services research
Secondary Subject Heading:	Health informatics, Emergency medicine, Health services research, Medical management, Research methods
Keywords:	computer aided-risk score, medical judgement, mortality, emergency medical admission

SCHOLARONE™ Manuscripts

A novel automated computer aided risk of mortality score is comparable to medical judgement in predicting a patient's risk of mortality following emergency medical admission: A prospective study of consecutive patients.

Authors

Muhammad Faisal, PhD
Senior Research Fellow in Biostatistics
Faculty of Health Studies, University of Bradford, Bradford, UK
Bradford Institute for Health Research
E-mail: M.Faisal1@bradford.ac.uk

Binish Khatoon, PhD
Research Fellow
Faculty of Health Studies, University of Bradford, Bradford, UK
Bradford Institute for Health Research
E-mail: B.Khatoon@bradford.ac.uk

Andy Scally, MSc
Senior Lecturer
School of Clinical Therapies
University College Cork, Ireland
E-mail: andrew.scally@ucc.ie

Donald Richardson, FRCP
Consultant Renal Physician
Department of Renal Medicine, York Teaching Hospital NHS Foundation Trust Hospital
E-mail: drichardson@doctors.org.uk

Sally Irwin FRCP
Consultant Geriatrician,
Department of Elderly Medicine,
York Teaching Hospital NHS Foundation Trust Hospital
Email:sally.irwin@york.nhs.uk

Rachel Davidson MRCP
Consultant Geriatrician,
Department of Elderly Medicine,
York Teaching Hospital NHS Foundation Trust Hospital
Email:Rachel.davidson@york.nhs.uk

David Heseltine FRCP
Consultant Geriatrician,
Department of Elderly Medicine,
York Teaching Hospital NHS Foundation Trust Hospital
Email:david.heseltine@york.nhs.uk

For peer review only - http://bmjopen.bmj.com/site/about/guidelines.xhtml

Alison Corlett FRCP
Consultant Geriatrician,
Department of Elderly Medicine,
York Teaching Hospital NHS Foundation Trust Hospital
Email: Alison.j.corlett@york.nhs.uk

Javed Ali MRCP (Ireland)
Consultant Geriatrician,
Department of Elderly Medicine,
York Teaching Hospital NHS Foundation Trust Hospital
Email:Javed.ali@york.nhs.uk

Rebecca Hampson MRCP
Consultant Geriatrician,
Department of Elderly Medicine,
York Teaching Hospital NHS Foundation Trust Hospital
Email: Rebecca.hampson@york.nhs.uk

Sandeep Kesavan FRCP (Edin)
Consultant Geriatrician,
Department of Elderly Medicine,
York Teaching Hospital NHS Foundation Trust Hospital
Email: Sandeep.kesavan@york.nhs.uk

Gerry McGonigal MD
Consultant Geriatrician,
Department of Elderly Medicine,
York Teaching Hospital NHS Foundation Trust Hospital
Email: Gerard.mcgonigal@york.nhs.uk

Karen Goodman FRCP (Edin)
Consultant Geriatrician,
Department of Elderly Medicine,
York Teaching Hospital NHS Foundation Trust Hospital
Email:Karen.goodman@york.nhs.uk

Michael Harkness FRCP
Consultant Geriatrician,
Department of Elderly Medicine,
York Teaching Hospital NHS Foundation Trust Hospital
Email: Michael.harkness@york.nhs.uk

Mohammed A Mohammed, PhD
Professor of Healthcare Quality & Effectiveness
Faculty of Health Studies, University of Bradford, Bradford, UK
The Strategy Unit, NHS Midlands and Lancashire Commissioning Support Unit
E-mail: M.A.Mohammed5@Bradford.ac.uk

Correspondence to Mohammed A Mohammed

For peer review only

For peer review only - http://bmjopen.bmj.com/site/about/guidelines.xhtml

Word-count: 2491

Abstract

Objective: To compare the performance of a validated automatic computer aided risk of mortality score (CARM) versus medical judgement in predicting the risk of in-hospital mortality for patients following emergency medical admission.

Method: Consecutive emergency admissions to an elderly care medical admissions ward in one hospital were assigned a risk of death at the first post take ward round by consultant staff over a two-week period. The same admissions were subsequently assigned a risk of death using the CARM score, based on age, sex, vital signs and blood test results. The performance of the CARM versus consultant medical judgement was compared using the c-statistic and the positive predictive value (PPV).

Results: The in-hospital mortality was 31.8% (130/409). For patients with complete blood test results, the c-statistic for CARM was 0.75 (95% CI 0.69 to 0.81) vs 0.72 (95% CI 0.66 to 0.78) for medical judgements (p=0.28). For patients with at least one missing blood test result the c-statistics were similar (medical judgements 0.70 (95%CI 0.60 to 0.81) vs CARM 0.70 (95%CI 0.59 to 0.80)). At a 10% mortality risk the PPV for CARM was higher than medical judgements in patients with complete blood test results 62.0% (95%CI 51.2 to 71.9) vs 49.2% (95%CI 39.8 to 58.5) but not when blood test results were missing 50.0% (95%CI 24.7 to 75.3) vs 53.3% (95%CI 34.3 to 71.7).

Conclusion: CARM is comparable with medical judgements in discriminating in-hospital mortality following emergency admission to an elderly care ward. CARM may have a promising role in supporting medical judgements in determining the patient's risk of death in hospital. Further evaluation of CARM in routine practice is required.

Keywords: computer aided-risk score; medical judgement; mortality; emergency medical admission

For peer review only

For peer review only - http://bmjopen.bmj.com/site/about/guidelines.xhtml

Article Summary

- This study compares a novel computer-aided risk of mortality (CARM) score versus medical judgement in predicting the risk of in-hospital mortality. CARM uses the patient's age, sex, vital signs and blood test results.
- Consecutive emergency admissions to an elderly care ward in one hospital were assigned a risk of death at the first post take ward round by consultant staff.
- We then compared the performance of CARM with consultant estimates of the patient's risk of dying in-hospital using the c-statistic
- For patients with complete blood test results CARM (c statistic 0.75) was comparable with medical judgment (c-statistic 0.72).
- For a ¼ of admissions with one or more blood test missing CARM (c statistic 0.70) was similar to medical judgment (c-statistic 0.70) with imputed blood test results.

For peer review only

For peer review only - http://bmjopen.bmj.com/site/about/guidelines.xhtml

Introduction

Over the past few decades, numerous scoring systems have been developed to estimate the risk of mortality in hospital settings including intensive care medicine emergency medicine [1] and to a lesser extent general medical wards [2]. Despite the preponderance of scoring systems, systematic reviews [2] have highlighted a lack robust evaluation of risk scoring systems and only a few studies [3–5] have assessed the their accuracy versus medical judgements in routine clinical settings. This is important because if the risk score is found not to perform well when compared to medical judgements, this would call into question the benefit of using the score in routine clinical practice. In a review of 12 studies in intensive care, Sinuff et al [6] found that physicians were better able to discriminate between survivors and non-survivors than scoring systems in the first 24 hours of admission. However one of their included studies [4] found that for patients at the extremes of risk of deterioration, clinicians outperformed scoring systems when assessing these patients but when assessing the "in-between" group of patients, scoring systems were better than clinical judgement [4].

We recently developed a computer aided risk of in-hospital mortality (CARM) score, which combines age, sex, vital signs (based on National Early Warning Score (NEWS) [7]) and seven blood test results for emergency medical admissions [8]. A key design feature of CARM is that it uses data which is already collected as part of the process of care and so places no additional data collection burden on clinicians. Furthermore, CARM is intended for computerised implementation and is not suited to pencil and paper methods because the underlying equation is not simple [9] as it involves 22 covariates with and without transformations and interaction effects. Nonetheless it is important to note that CARM is intended to support, not displace, clinical judgment but the extent to which it can support the clinical decision-making process in practice remains unknown. So, as part of the on-going evaluation of CARM we set out to compare the performance of CARM versus medical judgements in estimating the risk of in-hospital mortality in consecutive emergency admissions to elderly care wards in one hospital over a two-week period.

For peer review only - http://bmjopen.bmj.com/site/about/guidelines.xhtml

Methods

Setting & data

Our cohort of elderly medical admissions is from York Hospital (managed by York Teaching Hospitals NHS Foundation Trust) which has approximately 700 beds. It has been exclusively using electronic NEWS scoring since 2013 as part of their in-house electronic patient record systems. Consecutive admissions to an elderly care medical admissions ward in this hospital were assigned a risk of death at the first post take ward round by consultant medical staff over a two-week period (February 05, 2017 to February 20, 2017). The medical staff did not have access to the CARM score during the data collection exercise. The same admissions were subsequently assigned a risk of death using the CARM score, based on their age, sex, vital signs (based on NEWS) and blood test results [8]. For each admission, we obtained the patient's age, sex (male/female), admission and discharge date and time, AKI score, electronic National Early Warning Score (NEWS) (including its subcomponent vital signs data), and seven blood test results (albumin, creatinine, haemoglobin, potassium, sodium, urea, and white cell count), although not all patients have all seven blood tests. To derive a CARM score for patients with missing blood test results we imputed population-based age-sex median values. The reason for missing blood tests was that they were not ordered by the medical staff.

Statistical Analysis

The performance of CARM versus medical judgement was assessed by comparing risk estimates using boxplots. The discrimination of CARM and medical judgements was quantified by the area under the Receiver-Operating Characteristic (ROC) curve or c-statistic [10]. In general, values less than 0.7 are considered to show poor discrimination, values of 0.7 to 0.8 can be described as reasonable, and values above 0.8 suggest good discrimination [11]. We compared the c-statistic for CARM and medical judgement using DeLong's test [12].

We determined the sensitivity, specificity, positive and negative predictive values, and positive and negative likelihood ratios for CARM and compared this with medical judgement scores using

For peer review only - http://bmjopen.bmj.com/site/about/guidelines.xhtml

probability thresholds from a NEWS only model for NEWS scores from 1 to 5. The cut-off of NEWS at 5 is the recommended threshold for escalation of care [13,14]. We have also reported the geometric mean of sensitivity and specificity [15].

All analyses were undertaken in STATA [16] and R [17] using *rms* [18] and *pROC* [19] packages.

Ethical approval

This study received ethical approval from The Yorkshire & Humberside Leeds West Research Ethics Committee on 17 September 2015 (ref. 173753) with NHS management permissions received January 2016.

Patient and Public Involvement

A workshop with a patient and service user group, linked to the University of Bradford, was involved at the start of this project to co-design the agenda for the patient and staff focus groups which were subsequently held at each hospital site. Patients were invited to attend the patient focus group through existing patient and public involvement groups. The criteria used for recruitment to these focus groups was any member of the public who had been a patient or carer in the last five years. The patient and public voice continued to be included throughout the project with three patient representatives invited to sit on the project steering group. Participants will be informed of the results of this study through the patient and public involvement leads at each hospital site and the project team have met with the Bradford Patient and Service User Group to discuss the results.

For peer review only - http://bmjopen.bmj.com/site/about/guidelines.xhtml
For peer review only

Data Sharing Statement

Our data sharing agreement with the York Hospital does not permit us to share this data with other parties. Nonetheless if anyone is interested in the data, then they should contact the Research and Development offices at York Hospital in the first instance.

Results

Cohort description

The study involved 409 emergency medical admissions to the elderly care wards in York Hospital. Of these 300 (73.3%) had a full set of blood test and 109 (26.7%) had at least one blood test result missing (Table 1). The most frequent missing blood test was albumin (n=96).

Characteristic	Discharged alive (%)	Discharged deceased (%)	All (%)
Total emergency medical admissions	279	130	409
Complete blood test results recorded (%)	202 (72.4)	98 (75.4)	300 (73.3)
At least one blood test result is not recorded (%)	77 (27.6)	32 (24.6)	109 (26.7)

Table 1 Pattern of missing blood test results in discharged alive/deceased elderly medical admissions

The in-hospital mortality was 31.8% (130/409). The age, sex, NEWS and blood test results profile is shown in Table 2. Compared with patients discharged alive, deceased patients were aged older, with lower albumin, haemoglobin and sodium values, and higher creatinine, potassium, white cell count and urea values. NEWS was higher in deceased patients compared with patients discharged alive, as were respiratory rate and pulse rate values. The temperature, blood pressure and oxygen saturation values were lower in deceased patients. Where blood test results were missing we imputed the age-sex population median value which appeared to give more reasonable values for patients discharged alive than those who died (see imputed values in table 2 comparing imputed values with observed values). For example, the observed mean (n=313) for albumin is 36.7 for survivors vs 33.6 for non-

For peer review only - http://bmjopen.bmj.com/site/about/guidelines.xhtml

survivors. However, the imputed means for albumin (n=96) were 36.8 for survivors and 36.7 for non-survivors.

Characteristic	Discharged alive	Discharged deceased
N=409	279	130
Male (%)	123 (44.1)	68 (52.3)
Mean CARM Score (SD)	0.07 (0.07)	0.16 (0.16)
Mean Medical Judgement Risk Score (SD)	0.12 (0.14)	0.26 (0.25)
Mean NEWS (SD)	2 (2.0)	3.2 (3.2)
Alertness		
Alert (%)	278 (99.6)	123 (94.6)
Pain (%)	0 (0.0)	3 (2.3)
Voice (%)	1 (0.4)	4 (3.1)
Unconscious (%)	0 (0.0)	0 (0.0)
AKI Score		
0 (%)	271 (97.1)	122 (93.8)
1 (%)	5 (1.8)	5 (3.8)
2 (%)	2 (0.7)	2 (1.5)
3 (%)	1 (0.4)	1 (0.8)
Oxygen supplementation (%)	50 (17.9)	42 (32.3)
Mean Age [years] (SD)	84.4 (5.5)	86.7 (6.6)
Mean Respiratory rate [breaths per minute] (SD)	18.3 (2.9)	19.1 (4.4)
Mean Temperature [°C] (SD)	36.5 (0.7)	36.4 (0.8)
Mean Systolic pressure [mmHg] (SD)	135.8 (25)	124.1 (23.6)
Mean Diastolic pressure [mmHg] (SD)	71 (13.8)	68.2 (12.4)
Mean Pulse rate [beats per minute] (SD)	78.6 (16.4)	81.6 (18.3)
Mean % Oxygen saturation (SD)	96.1 (2)	95.5 (3.1)
Mean Albumin [g/L] (SD)		
- no imputation (n=313)	36.7 (4.3)	33.6 (5.8)
- with imputation (n=96) ƚ	36.8 (0.6)	36.7 (1.0)
Mean Creatinine [umol/L] (SD)		
- no imputation (n=391)	103.3 (59.2)	118.7 (75.3)
- with imputation (n=18) ƚ	91.7 (10.8)	88.7 (15.3)
Mean Haemoglobin [g/l] (SD)		
- no imputation (n=391)	123.3 (20.4)	117.8 (17.7)
- with imputation (n=18) ƚ	121.5 (4.4)	116.5 (5.0)
Mean Potassium [mmol/L] (SD)		
- no imputation (n=367)	4.3 (0.5)	4.4 (0.6)
- with imputation (n=42) ƚ	4.3 (0.1)	4.3 (0.1)
Mean Sodium [mmol/L] (SD)		
- no imputation (n=383)	136.1 (4.5)	135.5 (5.7)
- with imputation (n=26) ƚ	137.0 (0.4)	136.8 (0.4)
Mean White cell count [10^9 cells/L] (SD)		
- no imputation (n=391)	10.4 (6.4)	11.8 (12.8)
- with imputation (n=18) ƚ	9.2 (0.3)	9.25 (0.2)
Mean Urea [mmol/L] (SD)		
- no imputation (n=391)	9.2 (5.3)	12.3 (8.9)
- with imputation (n=18) ƚ	8.3 (0.8)	7.9 (1.4)

Table 2 Characteristics of all elderly medical admissions.

ƚ Imputed blood test results using age and sex specific population median values.

For peer review only - http://bmjopen.bmj.com/site/about/guidelines.xhtml

Comparison of CARM versus Medical Judgement

Figure 1 shows the estimated risk of in-hospital mortality using CARM versus medical judgements for patients who discharged alive and deceased. The mean estimated risk of in-hospital mortality for patients discharged alive was lower with CARM (0.07 SD=0.07) versus medical judgements (0.12 SD=0.14). Likewise, for decreased patients, the risk estimates from CARM (0.16 SD=0.16) were lower than estimates from medical judgements (0.26 SD=0.25) (see Table 2).

Figure 2 shows the ROC curve. The area under the ROC curve (c-statistic), was higher for CARM 0.75 (95% CI 0.69 to 0.81) than for medical judgement 0.72 (95% CI 0.66 to 0.78) and were not statistically significant (p-value = 0.28). The area under the ROC curve was similar for admissions with at least one blood test result missing (see Table 3).

**Imputation**	**Medical Judgement AUC (95% CI)**	**CARM AUC (95% CI)**	**p-value**
Complete blood test results (N=300)	0.72 (0.66 to 0.78)	0.75 (0.69 to 0.81)	0.28
At least one blood test result is imputed (N=109)	0.70 (0.60 to 0.81)	0.70 (0.59 to 0.80)	0.86

Table 3 Comparing discrimination of Medical Judgement versus CARM in predicting the risk of in-hospital mortality

AUC, area under the curve; CARM, computer-aided risk score for in-hospital mortality.

For peer review only

For peer review only - http://bmjopen.bmj.com/site/about/guidelines.xhtml

Table 4 shows the sensitivity, specificity, positive predictive value and negative predictive value for a selected range of NEWS values. For patients with complete blood test results (n=300), NEWS at 5 (the recommended escalation threshold), which is equivalent to a 10% risk of in-hospital mortality, medical judgement had a higher sensitivity 59.2% (95%CI 48.8 to 69.0) vs 58.2% (95%CI 47.8 to 68.1), lower specificity 70.3% (95%CI 63.5 to 76.5) vs 82.7% (95%CI 76.7 to 87.6), lower PPVs 49.2% (95%CI 39.8 to 58.5) vs 62.0% (95%CI 51.2 to 71.9) and a lower positive likelihood ratio (2 vs 3.4) than the CARM score.

For patients with at least one imputed blood test result (N=109), at a NEWS of 5 medical judgement had a higher sensitivity 50.0% (95%CI 31.9 to 68.1) vs 25.0% (95%CI 11.5 to 43.4), lower specificity 81.8% (95%CI 71.4 to 89.7) vs 89.6% (95%CI 80.6 to 95.4), higher PPVs 53.3% (95%CI 34.3 to 71.7) vs 50.0% (95%CI 24.7 to 75.3) and higher positive likelihood ratios (2.8 vs 2.4).

For peer review only - http://bmjopen.bmj.com/site/about/guidelines.xhtml

	NEWS	Predicted risk at NEWS thresholds	Medical Judgement								CARM							
			N	Sensitivity%	Specificity%	PPV	NPV	LR+	LR-	GM%	N	Sensitivity%	Specificity%	PPV	NPV	LR+	LR-	GM%
Complete Blood test results N=300	1	0.03	275	98.0 (92.8 to 99.8)	11.4 (7.4 to 16.6)	34.9 (29.3 to 40.9)	92.0 (74 to 99)	1.1 (1.0 to 1.2)	0.2 (0.0 to 0.7)	33.4	239	90.8 (83.3 to 95.7)	25.7 (19.9 to 32.3)	37.2 (31.1 to 43.7)	85.2 (73.8 to 93)	1.2 (1.1 to 1.4)	0.4 (0.2 to 0.7)	48.4
	2	0.04	273	98.0 (92.8 to 99.8)	12.4 (8.2 to 17.7)	35.2 (29.5 to 41.1)	92.6 (75.7 to 99.1)	1.1 (1.1 to 1.2)	0.2 (0.0 to 0.7)	34.8	205	84.7 (76 to 91.2)	39.6 (32.8 to 46.7)	40.5 (33.7 to 47.5)	84.2 (75.3 to 90.9)	1.4 (1.2 to 1.6)	0.4 (0.2 to 0.6)	57.9
	3	0.05	190	84.7 (76 to 91.2)	47.0 (40.0 to 54.2)	43.7 (36.5 to 51.1)	86.4 (78.5 to 92.2)	1.6 (1.4 to 1.9)	0.3 (0.2 to 0.5)	63.1	168	79.6 (70.3 to 87.1)	55.4 (48.3 to 62.4)	46.4 (38.7 to 54.3)	84.8 (77.6 to 90.5)	1.8 (1.5 to 2.1)	0.4 (0.2 to 0.6)	66.4
	4	0.08	186	83.7 (74.8 to 90.4)	48.5 (41.4 to 55.6)	44.1 (36.8 to 51.5)	86.0 (78.2 to 91.8)	1.6 (1.4 to 1.9)	0.3 (0.2 to 0.5)	63.7	126	65.3 (55.0 to 74.6)	69.3 (62.4 to 75.6)	50.8 (41.7 to 59.8)	80.5 (73.8 to 86.1)	2.1 (1.7 to 2.7)	0.5 (0.4 to 0.7)	67.3
	5	0.10	118	59.2 (48.8 to 69.0)	70.3 (63.5 to 76.5)	49.2 (39.8 to 58.5)	78.0 (71.3 to 83.8)	2.0 (1.5 to 2.6)	0.6 (0.5 to 0.7)	64.5	92	58.2 (47.8 to 68.1)	82.7 (76.7 to 87.6)	62.0 (51.2 to 71.9)	80.3 (74.2 to 85.5)	3.4 (2.4 to 4.7)	0.5 (0.4 to 0.6)	69.3
At least one blood test result is imputed N=109	1	0.03	89	93.8 (79.2 to 99.2)	23.4 (14.5 to 34.4)	33.7 (24.0 to 44.5)	90.0 (68.3 to 98.8)	1.2 (1.1 to 1.4)	0.3 (0.1 to 1.1)	46.8	83	90.6 (75.0 to 98.0)	29.9 (20.0 to 41.4)	34.9 (24.8 to 46.2)	88.5 (69.8 to 97.6)	1.3 (1.1 to 1.6)	0.3 (0.1 to 1.0)	52.0
	2	0.04	88	93.8 (79.2 to 99.2)	24.7 (15.6 to 35.8)	34.1 (24.3 to 45)	90.5 (69.6 to 98.8)	1.2 (1.1 to 1.5)	0.3 (0.1 to 1.0)	48.1	63	75.0 (56.6 to 88.5)	49.4 (37.8 to 61.0)	38.1 (26.1 to 51.2)	82.6 (68.6 to 92.2)	1.5 (1.1 to 2.0)	0.5 (0.3 to 1.0)	60.8
	3	0.05	59	68.8 (50.0 to 83.9)	51.9 (40.3 to 63.5)	37.3 (25.0 to 50.9)	80.0 (66.3 to 90.0)	1.4 (1.0 to 2.0)	0.6 (0.3 to 1.0)	59.8	47	62.5 (43.7 to 78.9)	64.9 (53.2 to 75.5)	42.6 (28.3 to 57.8)	80.6 (68.6 to 89.6)	1.8 (1.2 to 2.7)	0.6 (0.4 to 0.9)	63.7
	4	0.08	59	68.8 (50.0 to 83.9)	51.9 (40.3 to 63.5)	37.3 (25.0 to 50.9)	80.0 (66.3 to 90.0)	1.4 (1.0 to 2.0)	0.6 (0.3 to 1.0)	59.8	30	40.6 (23.7 to 59.4)	77.9 (67.0 to 86.6)	43.3 (25.5 to 62.6)	75.9 (65 to 84.9)	1.8 (1.0 to 3.3)	0.8 (0.6 to 1.0)	56.3
	5	0.10	30	50.0 (31.9 to 68.1)	81.8 (71.4 to 89.7)	53.3 (34.3 to 71.7)	79.7 (69.2 to 88.0)	2.8 (1.5 to 4.9)	0.6 (0.4 to 0.9)	64.0	16	25.0 (11.5 to 43.4)	89.6 (80.6 to 95.4)	50.0 (24.7 to 75.3)	74.2 (64.1 to 82.7)	2.4 (1.0 to 5.9)	0.8 (0.7 to 1.0)	47.3

Table 4 Performance of CARM versus medical judgement with/without imputation in predicting the risk in-hospital mortality at NEWS thresholds (1, 2, 3, 4, 5)

PPV=Positive Predictive Value; NPV= Negative Predictive Value; LR+=Positive Likelihood Ratio; LR-=Negative Likelihood Ratio; GM=geometric mean.

For peer review only

For peer review only - http://bmjopen.bmj.com/site/about/guidelines.xhtml

Discussion

In this study, we assessed the accuracy of CARM versus medical judgements in consecutive emergency admissions to the elderly care ward over a two-week period. We found for patients with complete blood test results, the c-statistic for CARM was 0.75 vs 0.72 for medical judgements (p=0.28). For patients with at least one missing blood test result the c-statistics were lower but still similar (medical judgements 0.70 vs CARM 0.70). At a 10% mortality risk the PPV for CARM was higher than medical judgements in patients with complete blood test results (62.0% vs 49.2%) but not when blood test results were missing (50.0% vs 53.3%).

Overall, when comparing CARM with medical judgements, no significant differences in AUC were found. These findings are remarkable because, unlike medical judgements, CARM relies exclusively on routinely collected data based primarily on the patients' age, vital signs and blood test results without having any disease labels or clinical history. Furthermore, where blood tests are being imputed CARS and medical judgements are less able to discriminate mortality. Whilst this is to be expected for CARM because we use a population median imputation strategy, which is biased towards survivors, the reasons for lower c-statistics for medical judgements is less clear. It would suggest that these patients (with one or more missing blood test results) are more challenging to assess for the medical staff although the underlying reasons are not clear.

Our findings are in line with other studies, which also found no significant differences between ROC curves for APACHE2 and clinical staff [20]. However, a study reported that the clinical assessment had an overall accuracy of 95.2% versus 90.9% for APACHE2 [3]. Other studies have also failed to show an advantage for the APACHE2 model when compared to medical judgements by the clinicians [4,5,21]. Another study found that physicians were significantly better in predicting outcome in a medical intensive care unit than APACHE [22]. One study concluded that physicians' clinical judgement could differ from scoring systems enough to account for large differences in expected outcomes [21].

For peer review only - http://bmjopen.bmj.com/site/about/guidelines.xhtml

It is important to note that we have designed CARM to support the medical decision-making process, not replace it, without placing any additional data collection burden on staff. The CARM risk prediction can also be made available as soon as the physiological observations and blood test results are available and prior to the consultant review which may be of assistance to more junior staff. CARM was developed using all adult non-elective medical and elderly care admissions to in one hospital and externally validated in another hospital [8].

The overall mortality was 5% in the study population in which the CARM risk predictor was developed. The overall mortality in this patient cohort is high and it is worth noting that patients had already been streamed (selected) as requiring in-patient admission as direct admission from GP or via the emergency department. Thus, the pre-test probability of mortality is different to original study population yet the CARM risk predictor still performs reasonably well in this population.

Our study has several limitations. This study provides a snapshot of the use of CARM in a hospital over a short period and the extent to which our findings generalise to patients over a longer time period and to other wards and hospitals requires further study. Although CARM is designed to be automated, we note that for 26% of patients were unable to derive the CARM score because of no or incomplete blood test results and the most frequent missing blood test result was albumin. Although we adopted a median imputation strategy the extent to which this is acceptable in routine clinical practice remains unknown especially as this imputation strategy is biased towards survivors and so will underestimate the true risk of dying for those who are likely to die. So further study is required to understand the issue of missing blood test results and how to address it in routine clinical practice. One possibility is that there may be an unintended increase in the use of blood test results in patients where blood test would not ordinarily be undertaken to simply provide a CARM score. Crucially how the medical decision-making process is modified by the availably or CARM and the extent to which it enhances situational awareness and subsequently enhances the quality of care without adverse unintended consequences remains to be seen.

For peer review only - http://bmjopen.bmj.com/site/about/guidelines.xhtml

Conclusions

CARM is comparable with medical judgements in predicting in-hospital mortality following emergency admission to an elderly care ward. CARM may have a promising role in supporting medical judgements in determining the patient's risk of death in hospital. Further evaluation of CARM in routine practice is required.

Funding

This research was supported by the Health Foundation. The Health Foundation is an independent charity working to improve the quality of health care in the UK.

This research was supported by the National Institute for Health Research (NIHR) Yorkshire and Humberside Patient Safety Translational Research Centre (NIHR YHPSTRC). The views expressed in this article are those of the author(s) and not necessarily those of the NHS, the NIHR, or the Department of Health and Social Care.

Contributorship

MAM & DR had the original idea for this work. MF undertook the statistical analyses with guidance from AS and MAM. DR gave a clinical perspective. MF and BK wrote the first draft of this paper. SI, RD, DH, AC, JA, RH, SK, GM, KG, MH contributed to data collection and all authors subsequently assisted in redrafting and have approved the final version. MAM will act as guarantor.

Competing Interests: The authors declare no conflicts of interest.

For peer review only

References

1 Brabrand M, Folkestad L, Clausen NG, *et al.* Risk scoring systems for adults admitted to the emergency department: a systematic review. *Scand J Trauma Resusc Emerg Med* 2010;**18**:8. doi:10.1186/1757-7241-18-8

2 Smith MEB, Chiovaro JC, O'Neil M, *et al.* Early Warning System Scores for Clinical Deterioration in Hospitalized Patients: A Systematic Review. *Ann Am Thorac Soc* 2014;**11**:1454–65. doi:10.1513/AnnalsATS.201403-102OC

3 Meyer AA, Messick WJ, Young P, *et al.* Prospective comparison of clinical judgment and APACHE II score in predicting the outcome in critically ill surgical patients. *J Trauma* 1992;**32**:744–7.http://europepmc.org/abstract/MED/1613834

4 McClish DK, Powell SH. How Well Can Physicians Estimate Mortality in a Medical Intensive Care Unit? *Med Decis Mak* 1989;**9**:125–32. doi:10.1177/0272989X8900900207

5 Christensen C, J Cottrell J, Murakami J, *et al. Forecasting Survival in the Medical Intensive Care Unit: A Comparison of Clinical Prognoses With Formal Estimates*. 1993. doi:10.1055/s-

For peer review only - http://bmjopen.bmj.com/site/about/guidelines.xhtml

0038-1634937
6 Sinuff T, Adhikari NKJ, Cook DJ, *et al.* Mortality predictions in the intensive care unit: Comparing physicians with scoring systems. *Crit Care Med* 2006;**34**:878–85. doi:10.1097/01.CCM.0000201881.58644.41
7 Royal College of Physicians. National Early Warning Score (NEWS): Standardising the assessment of acuteillness severity in the NHS - Report of a working party. 2012.
8 Faisal M, Scally A, Jackson N, *et al.* Development and validation of a novel computer-aided score to predict the risk of in-hospital mortality for acutely ill medical admissions in two acute hospitals using their first electronically recorded blood test results and vital signs: a cross-section. *BMJ Open (accepted Oct 2018)* 2018.
9 Faisal M, Scally AJ, Jackson N, *et al.* Supplementary data: Development and validation of a novel computer-aided score to predict the risk of in-hospital mortality for acutely ill medical admissions in two acute hospitals using their first electronically recorded blood test results and vital signs: a cross-sectional study. https://bmjopen.bmj.com/content/bmjopen/8/12/e022939/DC1/embed/inline-supplementary-material-1.pdf. *BMJ Open* 2018;**8**:e022939. doi:10.1136/bmjopen-2018-022939
10 Steyerberg EW, Vickers AJ, Cook NR, *et al.* Assessing the performance of prediction models: a framework for traditional and novel measures. *Epidemiology* 2010;**21**:128–38. doi:10.1097/EDE.0b013e3181c30fb2
11 Hanley JA, McNeil BJ. The meaning and use of the area under a receiver operating characteristic (ROC) curve. *Radiology* 1982;**143**:29–36. doi:10.1148/radiology.143.1.7063747
12 DeLong ER, DeLong DM, Clarke-Pearson DL. Comparing the areas under two or more correlated receiver operating characteristic curves: a nonparametric approach. *Biometrics* 1988;**44**:837–45.http://www.ncbi.nlm.nih.gov/pubmed/3203132 (accessed 4 Oct 2018).
13 Goulden R, Hoyle M-C, Monis J, *et al.* qSOFA, SIRS and NEWS for predicting inhospital mortality and ICU admission in emergency admissions treated as sepsis. *Emerg Med J* 2018;**35**:345–9. doi:10.1136/emermed-2017-207120
14 NHS. Royal College of Physicians: NHS England approves use of National Early Warning Score (NEWS) 2 to improve detection of acutely ill patients. 2017. https://www.rcplondon.ac.uk/news/nhs-england-approves-use-national-early-warning-score-news-2-improve-detection-acutely-ill
15 Sanz J, Paternain D, Galar M, *et al.* A new survival status prediction system for severe trauma patients based on a multiple classifier system. *Comput Methods Programs Biomed* 2017;**142**:1–8. doi:10.1016/j.cmpb.2017.02.011
16 StatCorp. Stata: Release 14. Statistical Software. College Station, TX: StataCorp LP. 2016.
17 R Development Core Team. R: A language and environment for statistical computing. R Foundation for Statistical Computing http://www.r-project.org/. 2015.
18 Harrell FE. rms: Regression Modeling Strategies http://cran.r-project.org/package=rms. 2015.
19 Robin X, Turck N, Hainard A, *et al.* pROC: an open-source package for R and S+ to analyze and compare ROC curves. *BMC Bioinformatics* 2011;**12**:77. doi:10.1186/1471-2105-12-77
20 Kruse JA, Thill-Baharozian MC, Carlson RW. Comparison of clinical assessment with APACHE II for predicting mortality risk in patients admitted to a medical intensive care unit. *JAMA* 1988;**260**:1739–42.
21 L. PG, M. PC. Physician risk assessment and apache scores in cardiac care units. *Clin Cardiol* 2009;**22**:366–8. doi:10.1002/clc.4960220514
22 Brannen AL 2nd, Godfrey LJ, Goetter WE. Prediction of outcome from critical illness. A comparison of clinical judgment with a prediction rule. *Arch Intern Med* 1989;**149**:1083–6.

For peer review only - http://bmjopen.bmj.com/site/about/guidelines.xhtml

Figure 1: Comparison of medical judgement versus CARM in predicting risk of mortality for patients who (A) discharged alive and (B) discharged deceased.

(A1/B1) Complete blood test results (N=300); **(A2/B2)** At least one blood test result is imputed (N=109)

Figure 2: Receiver Operating Characteristic curve for CARM and medical judgements with/ without imputed blood test results.

(A) Complete blood test results (N=300); **(B)** At least one blood test result is imputed (N=109)

Black line is for CARM and grey line is for medical judgement

For peer review only

For peer review only - http://bmjopen.bmj.com/site/about/guidelines.xhtml

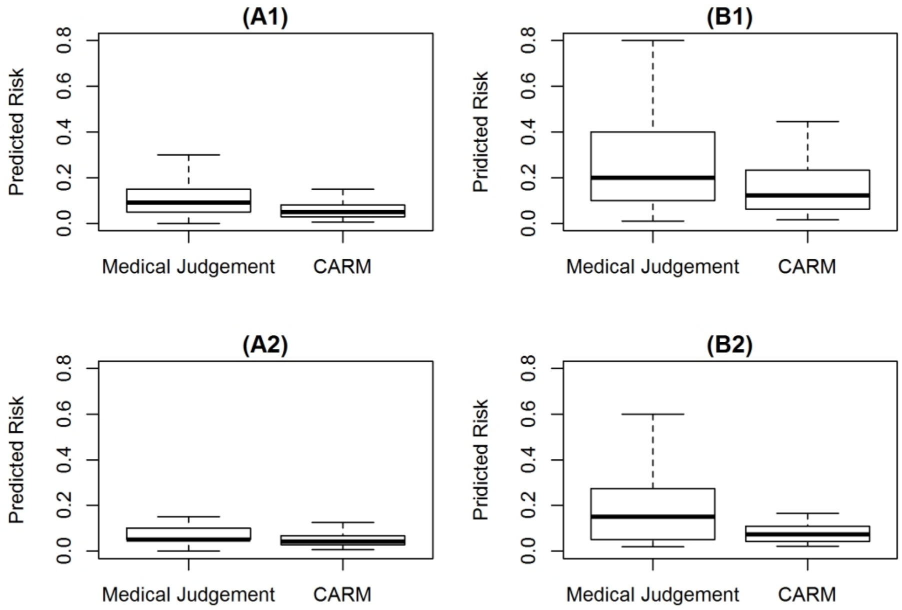

Comparison of medical judgement versus CARM in predicting risk of mortality for patients who (A) discharged alive and (B) discharged deceased.
(A1/B1) Complete blood test results (N=300); (A2/B2) At least one blood test result is imputed (N=109)

177x127mm (300 x 300 DPI)

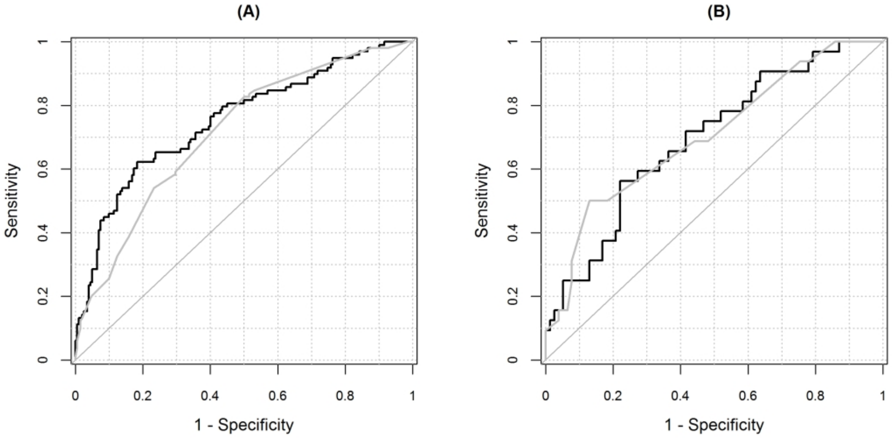

Receiver Operating Characteristic curve for CARM and medical judgements with/ without imputed blood test results.

(A) Complete blood test results (N=300); (B) At least one blood test result is imputed (N=109)
Black line is for CARM and grey line is for medical judgement

254x127mm (300 x 300 DPI)

STROBE Statement—checklist of items that should be included in reports of observational studies

	Item No	Recommendation	Pager number and comment
**Title and abstract**	1	(*a*) Indicate the study's design with a commonly used term in the title or the abstract	1
		(*b*) Provide in the abstract an informative and balanced summary of what was done and what was found	3-4
**Introduction**			
Background/rationale	2	Explain the scientific background and rationale for the investigation being reported	5
Objectives	3	State specific objectives, including any prespecified hypotheses	5
**Methods**			
Study design	4	Present key elements of study design early in the paper	6
Setting	5	Describe the setting, locations, and relevant dates, including periods of recruitment, exposure, follow-up, and data collection	6
Participants	6	(*a*) *Cohort study*—Give the eligibility criteria, and the sources and methods of selection of participants. Describe methods of follow-up<br>*Case-control study*—Give the eligibility criteria, and the sources and methods of case ascertainment and control selection. Give the rationale for the choice of cases and controls<br>*Cross-sectional study*—Give the eligibility criteria, and the sources and methods of selection of participants	6
		(*b*) *Cohort study*—For matched studies, give matching criteria and number of exposed and unexposed<br>*Case-control study*—For matched studies, give matching criteria and the number of controls per case	NA
Variables	7	Clearly define all outcomes, exposures, predictors, potential confounders, and effect modifiers. Give diagnostic criteria, if applicable	6
Data sources/ measurement	8*	For each variable of interest, give sources of data and details of methods of assessment (measurement). Describe comparability of assessment methods if there is more than one group	*6*
Bias	9	Describe any efforts to address potential sources of bias	6
Study size	10	Explain how the study size was arrived at	6
Quantitative variables	11	Explain how quantitative variables were handled in the analyses. If applicable, describe which groupings were chosen and why	6
Statistical methods	12	(*a*) Describe all statistical methods, including those used to control for confounding	
		(*b*) Describe any methods used to examine subgroups and interactions	
		(*c*) Explain how missing data were addressed	6
		(*d*) *Cohort study*—If applicable, explain how loss to follow-up was addressed<br>*Case-control study*—If applicable, explain how matching of cases and controls was addressed<br>*Cross-sectional study*—If applicable, describe analytical methods taking account of sampling strategy	NA
		(*e*) Describe any sensitivity analyses	6

Continued on next page

For peer review only - http://bmjopen.bmj.com/site/about/guidelines.xhtml

**Results**			
Participants	13*	(a) Report numbers of individuals at each stage of study—eg numbers potentially eligible, examined for eligibility, confirmed eligible, included in the study, completing follow-up, and analysed	8 (see table 1)
		(b) Give reasons for non-participation at each stage	8
		(c) Consider use of a flow diagram	NA
Descriptive data	14*	(a) Give characteristics of study participants (eg demographic, clinical, social) and information on exposures and potential confounders	9 (see table 2)
		(b) Indicate number of participants with missing data for each variable of interest	9
		(c) *Cohort study*—Summarise follow-up time (eg, average and total amount)	NA
Outcome data	15*	*Cohort study*—Report numbers of outcome events or summary measures over time	*NA*
		Case-control study—Report numbers in each exposure category, or summary measures of exposure	*NA*
		Cross-sectional study—Report numbers of outcome events or summary measures	9 (table 2)
Main results	16	(*a*) Give unadjusted estimates and, if applicable, confounder-adjusted estimates and their precision (eg, 95% confidence interval). Make clear which confounders were adjusted for and why they were included	NA
		(*b*) Report category boundaries when continuous variables were categorized	NA
		(*c*) If relevant, consider translating estimates of relative risk into absolute risk for a meaningful time period	NA
Other analyses	17	Report other analyses done—eg analyses of subgroups and interactions, and sensitivity analyses	10-12
**Discussion**			
Key results	18	Summarise key results with reference to study objectives	13
Limitations	19	Discuss limitations of the study, taking into account sources of potential bias or imprecision. Discuss both direction and magnitude of any potential bias	13-14
Interpretation	20	Give a cautious overall interpretation of results considering objectives, limitations, multiplicity of analyses, results from similar studies, and other relevant evidence	13-14
Generalisability	21	Discuss the generalisability (external validity) of the study results	13-14
**Other information**			
Funding	22	Give the source of funding and the role of the funders for the present study and, if applicable, for the original study on which the present article is based	15

*Give information separately for cases and controls in case-control studies and, if applicable, for exposed and unexposed groups in cohort and cross-sectional studies.

Note: An Explanation and Elaboration article discusses each checklist item and gives methodological background and published examples of transparent reporting. The STROBE checklist is best used in conjunction with this article (freely available on the Web sites of PLoS Medicine at http://www.plosmedicine.org/, Annals of Internal Medicine at http://www.annals.org/, and Epidemiology at http://www.epidem.com/). Information on the STROBE Initiative is available at www.strobe-statement.org.

For peer review only

For peer review only - http://bmjopen.bmj.com/site/about/guidelines.xhtml

BMJ Open

A prospective study of consecutive emergency medical admissions to compare a novel automated computer aided mortality risk score and clinical judgement of patient mortality risk.

Journal:	*BMJ Open*
Manuscript ID	bmjopen-2018-027741.R2
Article Type:	Research
Date Submitted by the Author:	28-May-2019
Complete List of Authors:	Faisal, Muhammad; University of Bradford,<br>Khatoon, Binish; University of Bradford Faculty of Health Studies<br>Scally, Andy; University College Cork National University of Ireland, School of Clinical Therapies<br>Richardson, Donald; York Teaching Hospital NHS Foundation Trust, Renal Unit<br>Irwin, Sally; York Teaching Hospital NHS Foundation Trust<br>Davidson, Rachel; York Teaching Hospital NHS Foundation Trust<br>Heseltine, David; York Teaching Hospital NHS Foundation Trust<br>Corlett, Alison; York Teaching Hospital NHS Foundation Trust<br>Ali, Javed; York Teaching Hospital NHS Foundation Trust<br>Hampson, Rebecca; York Teaching Hospital NHS Foundation Trust<br>Kesavan, Sandeep; York Teaching Hospital NHS Foundation Trust<br>McGonigal, Gerry; York Teaching Hospital NHS Foundation Trust<br>Goodman, Karen; York Teaching Hospital NHS Foundation Trust<br>Harkness, Michael; York Teaching Hospital NHS Foundation Trust<br>Mohammed, Mohammed ; University of Bradford Faculty of Health Studies; NHS Midlands and Lancashire Commissioning Support Unit
**Primary Subject Heading**:	Health services research
Secondary Subject Heading:	Health informatics, Emergency medicine, Health services research, Medical management, Research methods
Keywords:	computer aided-risk score, medical judgement, mortality, emergency medical admission

SCHOLARONE™
Manuscripts

A prospective study of consecutive emergency medical admissions to compare a novel automated computer aided mortality risk score and clinical judgement of patient mortality risk.

Authors

Muhammad Faisal, PhD
Senior Research Fellow in Biostatistics
Faculty of Health Studies, University of Bradford, Bradford, UK
Bradford Institute for Health Research
E-mail: M.Faisal1@bradford.ac.uk

Binish Khatoon, PhD
Research Fellow
Faculty of Health Studies, University of Bradford, Bradford, UK
Bradford Institute for Health Research
E-mail: B.Khatoon@bradford.ac.uk

Andy Scally, MSc
Senior Lecturer
School of Clinical Therapies
University College Cork, Ireland
E-mail: andrew.scally@ucc.ie

Donald Richardson, FRCP
Consultant Renal Physician
Department of Renal Medicine, York Teaching Hospital NHS Foundation Trust Hospital
E-mail: drichardson@doctors.org.uk

Sally Irwin FRCP
Consultant Geriatrician,
Department of Elderly Medicine,
York Teaching Hospital NHS Foundation Trust Hospital
Email:sally.irwin@york.nhs.uk

Rachel Davidson MRCP
Consultant Geriatrician,
Department of Elderly Medicine,
York Teaching Hospital NHS Foundation Trust Hospital
Email:Rachel.davidson@york.nhs.uk

David Heseltine FRCP
Consultant Geriatrician,
Department of Elderly Medicine,
York Teaching Hospital NHS Foundation Trust Hospital
Email:david.heseltine@york.nhs.uk

Alison Corlett FRCP
Consultant Geriatrician,

For peer review only - http://bmjopen.bmj.com/site/about/guidelines.xhtml

Department of Elderly Medicine,
York Teaching Hospital NHS Foundation Trust Hospital
Email: Alison.j.corlett@york.nhs.uk

Javed Ali MRCP (Ireland)
Consultant Geriatrician,
Department of Elderly Medicine,
York Teaching Hospital NHS Foundation Trust Hospital
Email:Javed.ali@york.nhs.uk

Rebecca Hampson MRCP
Consultant Geriatrician,
Department of Elderly Medicine,
York Teaching Hospital NHS Foundation Trust Hospital
Email: Rebecca.hampson@york.nhs.uk

Sandeep Kesavan FRCP (Edin)
Consultant Geriatrician,
Department of Elderly Medicine,
York Teaching Hospital NHS Foundation Trust Hospital
Email: Sandeep.kesavan@york.nhs.uk

Gerry McGonigal MD
Consultant Geriatrician,
Department of Elderly Medicine,
York Teaching Hospital NHS Foundation Trust Hospital
Email: Gerard.mcgonigal@york.nhs.uk

Karen Goodman FRCP (Edin)
Consultant Geriatrician,
Department of Elderly Medicine,
York Teaching Hospital NHS Foundation Trust Hospital
Email:Karen.goodman@york.nhs.uk

Michael Harkness FRCP
Consultant Geriatrician,
Department of Elderly Medicine,
York Teaching Hospital NHS Foundation Trust Hospital
Email: Michael.harkness@york.nhs.uk

Mohammed A Mohammed, PhD
Professor of Healthcare Quality & Effectiveness
Faculty of Health Studies, University of Bradford, Bradford, UK
The Strategy Unit, NHS Midlands and Lancashire Commissioning Support Unit
E-mail: M.A.Mohammed5@Bradford.ac.uk

Correspondence to Mohammed A Mohammed

Word-count: 2491

For peer review only

Abstract

Objectives: To compare the performance of a validated automatic computer aided risk of mortality score (CARM) versus medical judgement in predicting the risk of in-hospital mortality for patients following emergency medical admission.

Design: A prospective study

Setting: Consecutive emergency medical admissions in York hospital

Participants: Elderly medical admissions in one ward were assigned a risk of death at the first post take ward round by consultant staff over a two-week period. The consultant medical staff used the same variables to assign a risk of death to the patient as the CARM (age, sex, NEWS and blood test results) but also had access to the clinical history, examination findings and any immediately available investigations such as electrocardiograms (ECGs). The performance of the CARM versus consultant medical judgement was compared using the c-statistic and the positive predictive value (PPV).

Results: The in-hospital mortality was 31.8% (130/409). For patients with complete blood test results, the c-statistic for CARM was 0.75 (95% CI 0.69 to 0.81) vs 0.72 (95% CI 0.66 to 0.78) for medical judgements (p=0.28). For patients with at least one missing blood test result the c-statistics were similar (medical judgements 0.70 (95%CI 0.60 to 0.81) vs CARM 0.70 (95%CI 0.59 to 0.80)). At a 10% mortality risk the PPV for CARM was higher than medical judgements in patients with complete blood test results 62.0% (95%CI 51.2 to 71.9) vs 49.2% (95%CI 39.8 to 58.5) but not when blood test results were missing 50.0% (95%CI 24.7 to 75.3) vs 53.3% (95%CI 34.3 to 71.7).

Conclusions: CARM is comparable with medical judgements in discriminating in-hospital mortality following emergency admission to an elderly care ward. CARM may have a promising role in supporting medical judgements in determining the patient's risk of death in hospital. Further evaluation of CARM in routine practice is required.

Keywords: computer aided-risk score; medical judgement; mortality; emergency medical admission

For peer review only - http://bmjopen.bmj.com/site/about/guidelines.xhtml

Article Summary

Strengths and limitations

- This study compares a novel computer-aided risk of mortality (CARM) score versus medical judgement in predicting the risk of in-hospital mortality.
- Consecutive emergency admissions to an elderly care ward in one hospital were assigned a risk of death at the first post take ward round by consultant staff.
- The consultant medical staff used the same variables to assign a risk of death to the patient as the CARM (age, sex, NEWS and blood test results) but also had access to the clinical history, examination findings and any immediately available investigations such as electrocardiograms (ECGs).
- For a ¼ of admissions with one or more blood test missing CARM was similar to medical judgment with imputed blood test results.

For peer review only

For peer review only - http://bmjopen.bmj.com/site/about/guidelines.xhtml

Introduction

Over the past few decades, numerous scoring systems have been developed to estimate the risk of mortality in hospital settings including intensive care medicine emergency medicine [1] and to a lesser extent general medical wards [2]. Despite the preponderance of scoring systems, systematic reviews [2] have highlighted a lack robust evaluation of risk scoring systems and only a few studies [3–5] have assessed the their accuracy versus medical judgements in routine clinical settings. This is important because if the risk score is found not to perform well when compared to medical judgements, this would call into question the benefit of using the score in routine clinical practice. In a review of 12 studies in intensive care, Sinuff et al [6] found that physicians were better able to discriminate between survivors and non-survivors than scoring systems in the first 24 hours of admission. However one of their included studies [4] found that for patients at the extremes of risk of deterioration, clinicians outperformed scoring systems when assessing these patients but when assessing the "in-between" group of patients, scoring systems were better than clinical judgement [4].

We recently developed a computer aided risk of in-hospital mortality (CARM) score, which combines age, sex, vital signs (based on National Early Warning Score (NEWS) [7]) and seven blood test results for emergency medical admissions [8]. A key design feature of CARM is that it uses data which is already collected as part of the process of care and so places no additional data collection burden on clinicians. Furthermore, CARM is intended for computerised implementation and is not suited to pencil and paper methods because the underlying equation is not simple [9] as it involves 22 covariates with and without transformations and interaction effects. Nonetheless it is important to note that CARM is intended to support, not displace, clinical judgment but the extent to which it can support the clinical decision-making process in practice remains unknown. So, as part of the on-going evaluation of CARM we set out to compare the performance of CARM versus medical judgements in estimating the risk of in-hospital mortality in consecutive emergency admissions to elderly care wards in one hospital over a two-week period.

For peer review only - http://bmjopen.bmj.com/site/about/guidelines.xhtml

Methods

Setting & data

Our cohort of elderly medical admissions is from York Hospital (managed by York Teaching Hospitals NHS Foundation Trust) which has approximately 700 beds. It has been exclusively using electronic NEWS scoring since 2013 as part of their in-house electronic patient record systems. Consecutive admissions to an elderly care medical admissions ward in this hospital were assigned a risk of death at the first post take ward round by consultant medical staff over a two-week period (February 05, 2017 to February 20, 2017). The consultant medical staff used the same variables to assign a risk of death to the patient as the CARM (age, sex, NEWS and blood test results) [8] but also had access to the clinical history, examination findings and any immediately available investigations such as electrocardiograms (ECGs). Both, CARM and medical judgements had access to the same physiological and pathological variables. The medical staff did not have access to the CARM score during the data collection exercise. For each admission, we obtained the patient's age, sex (male/female), admission and discharge date and time, AKI score, electronic National Early Warning Score (NEWS) (including its subcomponent vital signs data), and seven blood test results (albumin, creatinine, haemoglobin, potassium, sodium, urea, and white cell count), although not all patients have all seven blood tests. To derive a CARM score for patients with missing blood test results we imputed population-based age-sex median values. The reason for missing blood tests was that they were not ordered by the medical staff.

Statistical Analysis

The performance of CARM versus medical judgement was assessed by comparing risk estimates using boxplots. The discrimination of CARM and medical judgements was quantified by the area under the Receiver-Operating Characteristic (ROC) curve or c-statistic [10]. In general, values less than 0.7 are considered to show poor discrimination, values of 0.7 to 0.8 can be described as reasonable, and

For peer review only - http://bmjopen.bmj.com/site/about/guidelines.xhtml

values above 0.8 suggest good discrimination [11]. We compared the c-statistic for CARM and medical judgement using DeLong's test [12].

We determined the sensitivity, specificity, positive and negative predictive values, and positive and negative likelihood ratios for CARM and compared this with medical judgement scores using probability thresholds from a NEWS only model for NEWS scores from 1 to 5. The cut-off of NEWS at 5 is the recommended threshold for escalation of care [13,14]. We have also reported the geometric mean of sensitivity and specificity [15].

All analyses were undertaken in STATA [16] and R [17] using *rms* [18] and *pROC* [19] packages.

Ethical approval

This study received ethical approval from The Yorkshire & Humberside Leeds West Research Ethics Committee on 17 September 2015 (ref. 173753) with NHS management permissions received January 2016.

Patient and Public Involvement

A workshop with a patient and service user group, linked to the University of Bradford, was involved at the start of this project to co-design the agenda for the patient and staff focus groups which were subsequently held at each hospital site. Patients were invited to attend the patient focus group through existing patient and public involvement groups. The criteria used for recruitment to these focus groups was any member of the public who had been a patient or carer in the last five years. The patient and public voice continued to be included throughout the project with three patient representatives invited to sit on the project steering group. Participants will be informed of the results of this study through the patient and public involvement leads at each hospital site and the project team have met with the Bradford Patient and Service User Group to discuss the results.

For peer review only - http://bmjopen.bmj.com/site/about/guidelines.xhtml

Results

Cohort description

The study involved 409 emergency medical admissions to the elderly care wards in York Hospital. Of these 300 (73.3%) had a full set of blood test and 109 (26.7%) had at least one blood test result missing (Table 1). The most frequent missing blood test was albumin (n=96).

Characteristic	Discharged alive (%)	Discharged deceased (%)	All (%)
Total emergency medical admissions	279	130	409
Complete blood test results recorded (%)	202 (72.4)	98 (75.4)	300 (73.3)
At least one blood test result is not recorded (%)	77 (27.6)	32 (24.6)	109 (26.7)

Table 1 Pattern of missing blood test results in discharged alive/deceased elderly medical admissions

The in-hospital mortality was 31.8% (130/409). The age, sex, NEWS and blood test results profile is shown in Table 2. Compared with patients discharged alive, deceased patients were aged older, with lower albumin, haemoglobin and sodium values, and higher creatinine, potassium, white cell count and urea values. NEWS was higher in deceased patients compared with patients discharged alive, as were respiratory rate and pulse rate values. The temperature, blood pressure and oxygen saturation values were lower in deceased patients. Where blood test results were missing we imputed the age-sex population median value which appeared to give more reasonable values for patients discharged alive than those who died (see imputed values in table 2 comparing imputed values with observed values). For example, the observed mean (n=313) for albumin is 36.7 for survivors vs 33.6 for non-survivors. However, the imputed means for albumin (n=96) were 36.8 for survivors and 36.7 for non-survivors.

Characteristic	Discharged alive	Discharged deceased
N=409	279	130
Male (%)	123 (44.1)	68 (52.3)
Mean CARM Score (SD)	0.07 (0.07)	0.16 (0.16)
Mean Medical Judgement Risk Score (SD)	0.12 (0.14)	0.26 (0.25)
Mean NEWS (SD)	2 (2.0)	3.2 (3.2)
Alertness		

For peer review only - http://bmjopen.bmj.com/site/about/guidelines.xhtml

Alert (%)	278 (99.6)	123 (94.6)
Pain (%)	0 (0.0)	3 (2.3)
Voice (%)	1 (0.4)	4 (3.1)
Unconscious (%)	0 (0.0)	0 (0.0)
AKI Score		
0 (%)	271 (97.1)	122 (93.8)
1 (%)	5 (1.8)	5 (3.8)
2 (%)	2 (0.7)	2 (1.5)
3 (%)	1 (0.4)	1 (0.8)
Oxygen supplementation (%)	50 (17.9)	42 (32.3)
Mean Age [years] (SD)	84.4 (5.5)	86.7 (6.6)
Mean Respiratory rate [breaths per minute] (SD)	18.3 (2.9)	19.1 (4.4)
Mean Temperature [ºC] (SD)	36.5 (0.7)	36.4 (0.8)
Mean Systolic pressure [mmHg] (SD)	135.8 (25)	124.1 (23.6)
Mean Diastolic pressure [mmHg] (SD)	71 (13.8)	68.2 (12.4)
Mean Pulse rate [beats per minute] (SD)	78.6 (16.4)	81.6 (18.3)
Mean % Oxygen saturation (SD)	96.1 (2)	95.5 (3.1)
Mean Albumin [g/L] (SD)		
- no imputation (n=313)	36.7 (4.3)	33.6 (5.8)
- with imputation (n=96) ǂ	36.8 (0.6)	36.7 (1.0)
Mean Creatinine [umol/L] (SD)		
- no imputation (n=391)	103.3 (59.2)	118.7 (75.3)
- with imputation (n=18) ǂ	91.7 (10.8)	88.7 (15.3)
Mean Haemoglobin [g/l] (SD)		
- no imputation (n=391)	123.3 (20.4)	117.8 (17.7)
- with imputation (n=18) ǂ	121.5 (4.4)	116.5 (5.0)
Mean Potassium [mmol/L] (SD)		
- no imputation (n=367)	4.3 (0.5)	4.4 (0.6)
- with imputation (n=42) ǂ	4.3 (0.1)	4.3 (0.1)
Mean Sodium [mmol/L] (SD)		
- no imputation (n=383)	136.1 (4.5)	135.5 (5.7)
- with imputation (n=26) ǂ	137.0 (0.4)	136.8 (0.4)
Mean White cell count [10^9 cells/L] (SD)		
- no imputation (n=391)	10.4 (6.4)	11.8 (12.8)
- with imputation (n=18) ǂ	9.2 (0.3)	9.25 (0.2)
Mean Urea [mmol/L] (SD)		
- no imputation (n=391)	9.2 (5.3)	12.3 (8.9)
- with imputation (n=18) ǂ	8.3 (0.8)	7.9 (1.4)

Table 2 Characteristics of all elderly medical admissions.

ǂ Imputed blood test results using age and sex specific population median values.

Comparison of CARM versus Medical Judgement

Figure 1 shows the estimated risk of in-hospital mortality using CARM versus medical judgements for patients who discharged alive and deceased. The mean estimated risk of in-hospital mortality for patients discharged alive was lower with CARM (0.07 SD=0.07) versus medical judgements (0.12

For peer review only - http://bmjopen.bmj.com/site/about/guidelines.xhtml

SD=0.14). Likewise, for decreased patients, the risk estimates from CARM (0.16 SD=0.16) were lower than estimates from medical judgements (0.26 SD=0.25) (see Table 2).

Figure 2 shows the ROC curve. The area under the ROC curve (c-statistic), was higher for CARM 0.75 (95% CI 0.69 to 0.81) than for medical judgement 0.72 (95% CI 0.66 to 0.78) and were not statistically significant (p-value = 0.28). The area under the ROC curve was similar for admissions with at least one blood test result missing (see Table 3).

**Imputation**	**Medical Judgement AUC (95% CI)**	**CARM AUC (95% CI)**	**p-value**
Complete blood test results (N=300)	0.72 (0.66 to 0.78)	0.75 (0.69 to 0.81)	0.28
At least one blood test result is imputed (N=109)	0.70 (0.60 to 0.81)	0.70 (0.59 to 0.80)	0.86

Table 3 Comparing discrimination of Medical Judgement versus CARM in predicting the risk of in-hospital mortality

AUC, area under the curve; CARM, computer-aided risk score for in-hospital mortality.

For peer review only

For peer review only - http://bmjopen.bmj.com/site/about/guidelines.xhtml

Table 4 shows the sensitivity, specificity, positive predictive value and negative predictive value for a selected range of NEWS values. For patients with complete blood test results (n=300), NEWS at 5 (the recommended escalation threshold), which is equivalent to a 10% risk of in-hospital mortality, medical judgement had a higher sensitivity 59.2% (95%CI 48.8 to 69.0) vs 58.2% (95%CI 47.8 to 68.1), lower specificity 70.3% (95%CI 63.5 to 76.5) vs 82.7% (95%CI 76.7 to 87.6), lower PPVs 49.2% (95%CI 39.8 to 58.5) vs 62.0% (95%CI 51.2 to 71.9) and a lower positive likelihood ratio (2 vs 3.4) than the CARM score.

For patients with at least one imputed blood test result (N=109), at a NEWS of 5 medical judgement had a higher sensitivity 50.0% (95%CI 31.9 to 68.1) vs 25.0% (95%CI 11.5 to 43.4), lower specificity 81.8% (95%CI 71.4 to 89.7) vs 89.6% (95%CI 80.6 to 95.4), higher PPVs 53.3% (95%CI 34.3 to 71.7) vs 50.0% (95%CI 24.7 to 75.3) and higher positive likelihood ratios (2.8 vs 2.4).

For peer review only - http://bmjopen.bmj.com/site/about/guidelines.xhtml

	NEWS	Predicted risk at NEWS thresholds	Medical Judgement								CARM							
			N	Sensitivity%	Specificity%	PPV	NPV	LR+	LR-	GM%	N	Sensitivity%	Specificity%	PPV	NPV	LR+	LR-	GM%
Complete Blood test results N=300	1	0.03	275	98.0 (92.8 to 99.8)	11.4 (7.4 to 16.6)	34.9 (29.3 to 40.9)	92.0 (74 to 99)	1.1 (1.0 to 1.2)	0.2 (0.0 to 0.7)	33.4	239	90.8 (83.3 to 95.7)	25.7 (19.9 to 32.3)	37.2 (31.1 to 43.7)	85.2 (73.8 to 93)	1.2 (1.1 to 1.4)	0.4 (0.2 to 0.7)	48.4
	2	0.04	273	98.0 (92.8 to 99.8)	12.4 (8.2 to 17.7)	35.2 (29.5 to 41.1)	92.6 (75.7 to 99.1)	1.1 (1.1 to 1.2)	0.2 (0.0 to 0.7)	34.8	205	84.7 (76 to 91.2)	39.6 (32.8 to 46.7)	40.5 (33.7 to 47.5)	84.2 (75.3 to 90.9)	1.4 (1.2 to 1.6)	0.4 (0.2 to 0.6)	57.9
	3	0.05	190	84.7 (76 to 91.2)	47.0 (40.0 to 54.2)	43.7 (36.5 to 51.1)	86.4 (78.5 to 92.2)	1.6 (1.4 to 1.9)	0.3 (0.2 to 0.5)	63.1	168	79.6 (70.3 to 87.1)	55.4 (48.3 to 62.4)	46.4 (38.7 to 54.3)	84.8 (77.6 to 90.5)	1.8 (1.5 to 2.1)	0.4 (0.2 to 0.6)	66.4
	4	0.08	186	83.7 (74.8 to 90.4)	48.5 (41.4 to 55.6)	44.1 (36.8 to 51.5)	86.0 (78.2 to 91.8)	1.6 (1.4 to 1.9)	0.3 (0.2 to 0.5)	63.7	126	65.3 (55.0 to 74.6)	69.3 (62.4 to 75.6)	50.8 (41.7 to 59.8)	80.5 (73.8 to 86.1)	2.1 (1.7 to 2.7)	0.5 (0.4 to 0.7)	67.3
	5	0.10	118	59.2 (48.8 to 69.0)	70.3 (63.5 to 76.5)	49.2 (39.8 to 58.5)	78.0 (71.3 to 83.8)	2.0 (1.5 to 2.6)	0.6 (0.5 to 0.7)	64.5	92	58.2 (47.8 to 68.1)	82.7 (76.7 to 87.6)	62.0 (51.2 to 71.9)	80.3 (74.2 to 85.5)	3.4 (2.4 to 4.7)	0.5 (0.4 to 0.6)	69.3
At least one blood test result is imputed N=109	1	0.03	89	93.8 (79.2 to 99.2)	23.4 (14.5 to 34.4)	33.7 (24.0 to 44.5)	90.0 (68.3 to 98.8)	1.2 (1.1 to 1.4)	0.3 (0.1 to 1.1)	46.8	83	90.6 (75.0 to 98.0)	29.9 (20.0 to 41.4)	34.9 (24.8 to 46.2)	88.5 (69.8 to 97.6)	1.3 (1.1 to 1.6)	0.3 (0.1 to 1.0)	52.0
	2	0.04	88	93.8 (79.2 to 99.2)	24.7 (15.6 to 35.8)	34.1 (24.3 to 45)	90.5 (69.6 to 98.8)	1.2 (1.1 to 1.5)	0.3 (0.1 to 1.0)	48.1	63	75.0 (56.6 to 88.5)	49.4 (37.8 to 61.0)	38.1 (26.1 to 51.2)	82.6 (68.6 to 92.2)	1.5 (1.1 to 2.0)	0.5 (0.3 to 1.0)	60.8
	3	0.05	59	68.8 (50.0 to 83.9)	51.9 (40.3 to 63.5)	37.3 (25.0 to 50.9)	80.0 (66.3 to 90.0)	1.4 (1.0 to 2.0)	0.6 (0.3 to 1.0)	59.8	47	62.5 (43.7 to 78.9)	64.9 (53.2 to 75.5)	42.6 (28.3 to 57.8)	80.6 (68.6 to 89.6)	1.8 (1.2 to 2.7)	0.6 (0.4 to 0.9)	63.7
	4	0.08	59	68.8 (50.0 to 83.9)	51.9 (40.3 to 63.5)	37.3 (25.0 to 50.9)	80.0 (66.3 to 90.0)	1.4 (1.0 to 2.0)	0.6 (0.3 to 1.0)	59.8	30	40.6 (23.7 to 59.4)	77.9 (67.0 to 86.6)	43.3 (25.5 to 62.6)	75.9 (65 to 84.9)	1.8 (1.0 to 3.3)	0.8 (0.6 to 1.0)	56.3
	5	0.10	30	50.0 (31.9 to 68.1)	81.8 (71.4 to 89.7)	53.3 (34.3 to 71.7)	79.7 (69.2 to 88.0)	2.8 (1.5 to 4.9)	0.6 (0.4 to 0.9)	64.0	16	25.0 (11.5 to 43.4)	89.6 (80.6 to 95.4)	50.0 (24.7 to 75.3)	74.2 (64.1 to 82.7)	2.4 (1.0 to 5.9)	0.8 (0.7 to 1.0)	47.3

Table 4 Performance of CARM versus medical judgement with/without imputation in predicting the risk in-hospital mortality at NEWS thresholds (1, 2, 3, 4, 5)

PPV=Positive Predictive Value; NPV= Negative Predictive Value; LR+=Positive Likelihood Ratio; LR-=Negative Likelihood Ratio; GM=geometric mean.

For peer review only - http://bmjopen.bmj.com/site/about/guidelines.xhtml

For peer review only

Discussion

In this study, we assessed the accuracy of CARM versus medical judgements in consecutive emergency admissions to the elderly care ward over a two-week period. We found for patients with complete blood test results, the c-statistic for CARM was 0.75 vs 0.72 for medical judgements (p=0.28). For patients with at least one missing blood test result the c-statistics were lower but still similar (medical judgements 0.70 vs CARM 0.70). At a 10% mortality risk the PPV for CARM was higher than medical judgements in patients with complete blood test results (62.0% vs 49.2%) but not when blood test results were missing (50.0% vs 53.3%).

Overall, when comparing CARM with medical judgements, no significant differences in AUC were found. These findings are remarkable because, unlike medical judgements, CARM relies exclusively on routinely collected data based primarily on the patients' age, vital signs and blood test results without having any disease labels or clinical history. Furthermore, where blood tests are being imputed CARS and medical judgements are less able to discriminate mortality. Whilst this is to be expected for CARM because we use a population median imputation strategy, which is biased towards survivors, the reasons for lower c-statistics for medical judgements is less clear. It would suggest that these patients (with one or more missing blood test results) are more challenging to assess for the medical staff although the underlying reasons are not clear.

Our findings are in line with other studies, which also found no significant differences between ROC curves for APACHE2 and clinical staff [20]. However, a study reported that the clinical assessment had an overall accuracy of 95.2% versus 90.9% for APACHE2 [3]. Other studies have also failed to show an advantage for the APACHE2 model when compared to medical judgements by the clinicians [4,5,21]. Another study found that physicians were significantly better in predicting outcome in a medical intensive care unit than APACHE [22]. One study concluded that physicians' clinical judgement could differ from scoring systems enough to account for large differences in expected outcomes [21].

For peer review only - http://bmjopen.bmj.com/site/about/guidelines.xhtml

It is important to note that we have designed CARM to support the medical decision-making process, not replace it, without placing any additional data collection burden on staff. The CARM risk prediction can also be made available as soon as the physiological observations and blood test results are available and prior to the consultant review which may be of assistance to more junior staff. CARM was developed using all adult non-elective medical and elderly care admissions to in one hospital and externally validated in another hospital [8].

The overall mortality was 5% in the study population in which the CARM risk predictor was developed. The overall mortality in this patient cohort is high and it is worth noting that patients had already been streamed (selected) as requiring in-patient admission as direct admission from GP or via the emergency department. Thus, the pre-test probability of mortality is different to original study population yet the CARM risk predictor still performs reasonably well in this population.

Our study has several limitations. This study provides a snapshot of the use of CARM in a hospital over a short period and the extent to which our findings generalise to patients over a longer time period and to other wards and hospitals requires further study. Although CARM is designed to be automated, we note that for 26% of patients were unable to derive the CARM score because of no or incomplete blood test results and the most frequent missing blood test result was albumin. Although we adopted a median imputation strategy the extent to which this is acceptable in routine clinical practice remains unknown especially as this imputation strategy is biased towards survivors and so will underestimate the true risk of dying for those who are likely to die. So further study is required to understand the issue of missing blood test results and how to address it in routine clinical practice. One possibility is that there may be an unintended increase in the use of blood test results in patients where blood test would not ordinarily be undertaken to simply provide a CARM score. Crucially how the medical decision-making process is modified by the availably or CARM and the extent to which it enhances situational awareness and subsequently enhances the quality of care without adverse unintended consequences remains to be seen.

For peer review only - http://bmjopen.bmj.com/site/about/guidelines.xhtml

For peer review only

Conclusions

CARM is comparable with medical judgements in predicting in-hospital mortality following emergency admission to an elderly care ward. CARM may have a promising role in supporting medical judgements in determining the patient's risk of death in hospital. Further evaluation of CARM in routine practice is required.

Data Sharing Statement

Our data sharing agreement with the York Hospital does not permit us to share this data with other parties. Nonetheless if anyone is interested in the data, then they should contact the Research and Development offices at York Hospital in the first instance.

Funding

This research was supported by the Health Foundation. The Health Foundation is an independent charity working to improve the quality of health care in the UK.

This research was supported by the National Institute for Health Research (NIHR) Yorkshire and Humberside Patient Safety Translational Research Centre (NIHR YHPSTRC). The views expressed in this article are those of the author(s) and not necessarily those of the NHS, the NIHR, or the Department of Health and Social Care.

Contributorship

MAM & DR had the original idea for this work. MF undertook the statistical analyses with guidance from AS and MAM. DR gave a clinical perspective. MF and BK wrote the first draft of this paper. SI, RD, DH, AC, JA, RH, SK, GM, KG, MH contributed to data collection and all authors subsequently assisted in redrafting and have approved the final version. MAM will act as guarantor.

Competing Interests: The authors declare no conflicts of interest.

References

1 Brabrand M, Folkestad L, Clausen NG, *et al.* Risk scoring systems for adults admitted to the emergency department: a systematic review. *Scand J Trauma Resusc Emerg Med* 2010;**18**:8. doi:10.1186/1757-7241-18-8

2 Smith MEB, Chiovaro JC, O'Neil M, *et al.* Early Warning System Scores for Clinical

For peer review only - http://bmjopen.bmj.com/site/about/guidelines.xhtml

Deterioration in Hospitalized Patients: A Systematic Review. *Ann Am Thorac Soc* 2014;**11**:1454–65. doi:10.1513/AnnalsATS.201403-102OC
3 Meyer AA, Messick WJ, Young P, *et al.* Prospective comparison of clinical judgment and APACHE II score in predicting the outcome in critically ill surgical patients. *J Trauma* 1992;**32**:744–7.http://europepmc.org/abstract/MED/1613834
4 McClish DK, Powell SH. How Well Can Physicians Estimate Mortality in a Medical Intensive Care Unit? *Med Decis Mak* 1989;**9**:125–32. doi:10.1177/0272989X8900900207
5 Christensen C, J Cottrell J, Murakami J, *et al. Forecasting Survival in the Medical Intensive Care Unit: A Comparison of Clinical Prognoses With Formal Estimates*. 1993. doi:10.1055/s-0038-1634937
6 Sinuff T, Adhikari NKJ, Cook DJ, *et al.* Mortality predictions in the intensive care unit: Comparing physicians with scoring systems. *Crit Care Med* 2006;**34**:878–85. doi:10.1097/01.CCM.0000201881.58644.41
7 Royal College of Physicians. National Early Warning Score (NEWS): Standardising the assessment of acuteillness severity in the NHS - Report of a working party. 2012.
8 Faisal M, Scally A, Jackson N, *et al.* Development and validation of a novel computer-aided score to predict the risk of in-hospital mortality for acutely ill medical admissions in two acute hospitals using their first electronically recorded blood test results and vital signs: a cross-section. *BMJ Open (accepted Oct 2018)* 2018.
9 Faisal M, Scally AJ, Jackson N, *et al.* Supplementary data: Development and validation of a novel computer-aided score to predict the risk of in-hospital mortality for acutely ill medical admissions in two acute hospitals using their first electronically recorded blood test results and vital signs: a cross-sectional study. https://bmjopen.bmj.com/content/bmjopen/8/12/e022939/DC1/embed/inline-supplementary-material-1.pdf. *BMJ Open* 2018;**8**:e022939. doi:10.1136/bmjopen-2018-022939
10 Steyerberg EW, Vickers AJ, Cook NR, *et al.* Assessing the performance of prediction models: a framework for traditional and novel measures. *Epidemiology* 2010;**21**:128–38. doi:10.1097/EDE.0b013e3181c30fb2
11 Hanley JA, McNeil BJ. The meaning and use of the area under a receiver operating characteristic (ROC) curve. *Radiology* 1982;**143**:29–36. doi:10.1148/radiology.143.1.7063747
12 DeLong ER, DeLong DM, Clarke-Pearson DL. Comparing the areas under two or more correlated receiver operating characteristic curves: a nonparametric approach. *Biometrics* 1988;**44**:837–45.http://www.ncbi.nlm.nih.gov/pubmed/3203132 (accessed 4 Oct 2018).
13 Goulden R, Hoyle M-C, Monis J, *et al.* qSOFA, SIRS and NEWS for predicting inhospital mortality and ICU admission in emergency admissions treated as sepsis. *Emerg Med J* 2018;**35**:345–9. doi:10.1136/emermed-2017-207120
14 NHS. Royal College of Physicians: NHS England approves use of National Early Warning Score (NEWS) 2 to improve detection of acutely ill patients. 2017. https://www.rcplondon.ac.uk/news/nhs-england-approves-use-national-early-warning-score-news-2-improve-detection-acutely-ill
15 Sanz J, Paternain D, Galar M, *et al.* A new survival status prediction system for severe trauma patients based on a multiple classifier system. *Comput Methods Programs Biomed* 2017;**142**:1–8. doi:10.1016/j.cmpb.2017.02.011
16 StatCorp. Stata: Release 14. Statistical Software. College Station, TX: StataCorp LP. 2016.
17 R Development Core Team. R: A language and environment for statistical computing. R Foundation for Statistical Computing http://www.r-project.org/. 2015.
18 Harrell FE. rms: Regression Modeling Strategies http://cran.r-project.org/package=rms. 2015.
19 Robin X, Turck N, Hainard A, *et al.* pROC: an open-source package for R and S+ to analyze and compare ROC curves. *BMC Bioinformatics* 2011;**12**:77. doi:10.1186/1471-2105-12-77
20 Kruse JA, Thill-Baharozian MC, Carlson RW. Comparison of clinical assessment with APACHE II

For peer review only - http://bmjopen.bmj.com/site/about/guidelines.xhtml

for predicting mortality risk in patients admitted to a medical intensive care unit. *JAMA* 1988;**260**:1739–42.
21 L. PG, M. PC. Physician risk assessment and apache scores in cardiac care units. *Clin Cardiol* 2009;**22**:366–8. doi:10.1002/clc.4960220514
22 Brannen AL 2nd, Godfrey LJ, Goetter WE. Prediction of outcome from critical illness. A comparison of clinical judgment with a prediction rule. *Arch Intern Med* 1989;**149**:1083–6.

For peer review only

For peer review only - http://bmjopen.bmj.com/site/about/guidelines.xhtml

Figure 1: Comparison of medical judgement versus CARM in predicting risk of mortality for patients who (A) discharged alive and (B) discharged deceased.

(A1/B1) Complete blood test results (N=300); **(A2/B2)** At least one blood test result is imputed (N=109)

Figure 2: Receiver Operating Characteristic curve for CARM and medical judgements with/ without imputed blood test results.

(A) Complete blood test results (N=300); **(B)** At least one blood test result is imputed (N=109)

Black line is for CARM and grey line is for medical judgement

For peer review only

For peer review only - http://bmjopen.bmj.com/site/about/guidelines.xhtml

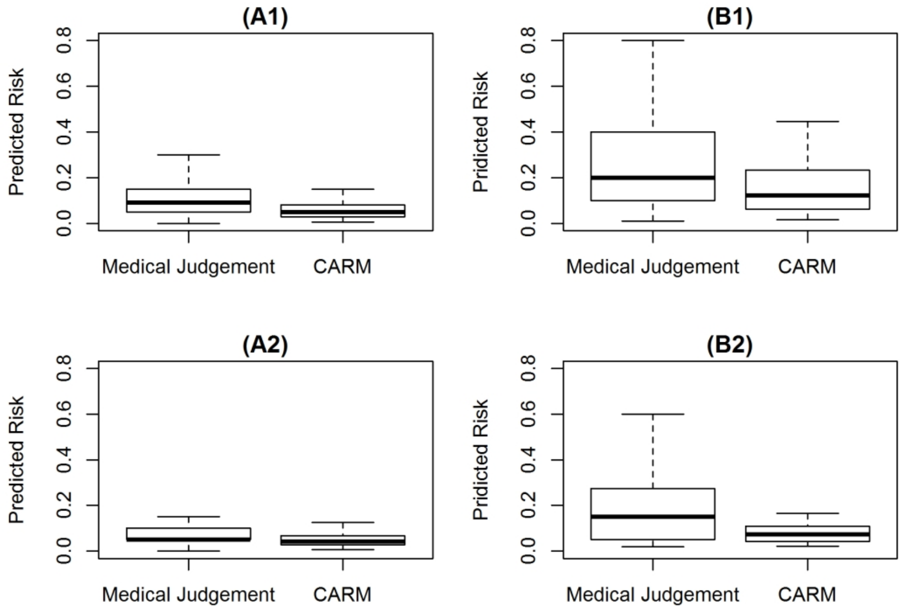

Comparison of medical judgement versus CARM in predicting risk of mortality for patients who (A) discharged alive and (B) discharged deceased.
(A1/B1) Complete blood test results (N=300); (A2/B2) At least one blood test result is imputed (N=109)

177x127mm (300 x 300 DPI)

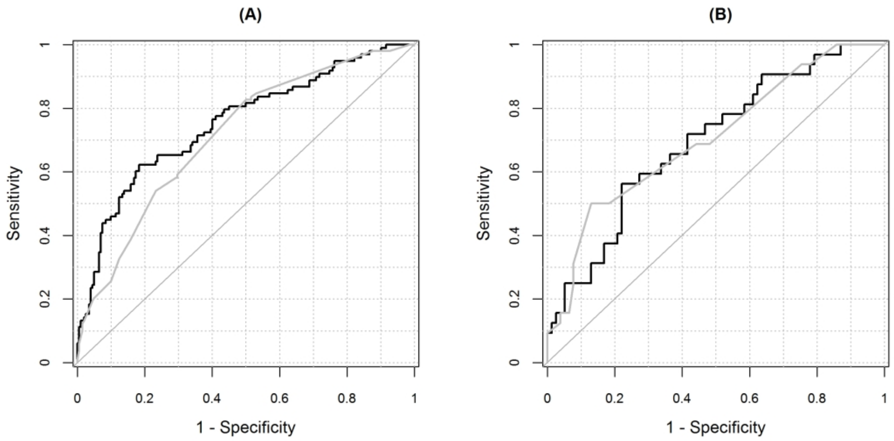

Receiver Operating Characteristic curve for CARM and medical judgements with/ without imputed blood test results.

(A) Complete blood test results (N=300); (B) At least one blood test result is imputed (N=109)
Black line is for CARM and grey line is for medical judgement

254x127mm (300 x 300 DPI)

STROBE Statement—checklist of items that should be included in reports of observational studies

	Item No	Recommendation	Pager number and comment
**Title and abstract**	1	(*a*) Indicate the study's design with a commonly used term in the title or the abstract	1
		(*b*) Provide in the abstract an informative and balanced summary of what was done and what was found	3-4
**Introduction**			
Background/rationale	2	Explain the scientific background and rationale for the investigation being reported	5
Objectives	3	State specific objectives, including any prespecified hypotheses	5
**Methods**			
Study design	4	Present key elements of study design early in the paper	6
Setting	5	Describe the setting, locations, and relevant dates, including periods of recruitment, exposure, follow-up, and data collection	6
Participants	6	(*a*) *Cohort study*—Give the eligibility criteria, and the sources and methods of selection of participants. Describe methods of follow-up<br>*Case-control study*—Give the eligibility criteria, and the sources and methods of case ascertainment and control selection. Give the rationale for the choice of cases and controls<br>*Cross-sectional study*—Give the eligibility criteria, and the sources and methods of selection of participants	6
		(*b*) *Cohort study*—For matched studies, give matching criteria and number of exposed and unexposed<br>*Case-control study*—For matched studies, give matching criteria and the number of controls per case	NA
Variables	7	Clearly define all outcomes, exposures, predictors, potential confounders, and effect modifiers. Give diagnostic criteria, if applicable	6
Data sources/ measurement	8*	For each variable of interest, give sources of data and details of methods of assessment (measurement). Describe comparability of assessment methods if there is more than one group	*6*
Bias	9	Describe any efforts to address potential sources of bias	6
Study size	10	Explain how the study size was arrived at	6
Quantitative variables	11	Explain how quantitative variables were handled in the analyses. If applicable, describe which groupings were chosen and why	6
Statistical methods	12	(*a*) Describe all statistical methods, including those used to control for confounding	
		(*b*) Describe any methods used to examine subgroups and interactions	
		(*c*) Explain how missing data were addressed	6
		(*d*) *Cohort study*—If applicable, explain how loss to follow-up was addressed<br>*Case-control study*—If applicable, explain how matching of cases and controls was addressed<br>*Cross-sectional study*—If applicable, describe analytical methods taking account of sampling strategy	NA
		(*e*) Describe any sensitivity analyses	6

Continued on next page

For peer review only - http://bmjopen.bmj.com/site/about/guidelines.xhtml

**Results**			
Participants	13*	(a) Report numbers of individuals at each stage of study—eg numbers potentially eligible, examined for eligibility, confirmed eligible, included in the study, completing follow-up, and analysed	8 (see table 1)
		(b) Give reasons for non-participation at each stage	8
		(c) Consider use of a flow diagram	NA
Descriptive data	14*	(a) Give characteristics of study participants (eg demographic, clinical, social) and information on exposures and potential confounders	9 (see table 2)
		(b) Indicate number of participants with missing data for each variable of interest	9
		(c) *Cohort study*—Summarise follow-up time (eg, average and total amount)	NA
Outcome data	15*	*Cohort study*—Report numbers of outcome events or summary measures over time	*NA*
		Case-control study—Report numbers in each exposure category, or summary measures of exposure	*NA*
		Cross-sectional study—Report numbers of outcome events or summary measures	9 (table 2)
Main results	16	(*a*) Give unadjusted estimates and, if applicable, confounder-adjusted estimates and their precision (eg, 95% confidence interval). Make clear which confounders were adjusted for and why they were included	NA
		(*b*) Report category boundaries when continuous variables were categorized	NA
		(*c*) If relevant, consider translating estimates of relative risk into absolute risk for a meaningful time period	NA
Other analyses	17	Report other analyses done—eg analyses of subgroups and interactions, and sensitivity analyses	10-12
**Discussion**			
Key results	18	Summarise key results with reference to study objectives	13
Limitations	19	Discuss limitations of the study, taking into account sources of potential bias or imprecision. Discuss both direction and magnitude of any potential bias	13-14
Interpretation	20	Give a cautious overall interpretation of results considering objectives, limitations, multiplicity of analyses, results from similar studies, and other relevant evidence	13-14
Generalisability	21	Discuss the generalisability (external validity) of the study results	13-14
**Other information**			
Funding	22	Give the source of funding and the role of the funders for the present study and, if applicable, for the original study on which the present article is based	15

*Give information separately for cases and controls in case-control studies and, if applicable, for exposed and unexposed groups in cohort and cross-sectional studies.

Note: An Explanation and Elaboration article discusses each checklist item and gives methodological background and published examples of transparent reporting. The STROBE checklist is best used in conjunction with this article (freely available on the Web sites of PLoS Medicine at http://www.plosmedicine.org/, Annals of Internal Medicine at http://www.annals.org/, and Epidemiology at http://www.epidem.com/). Information on the STROBE Initiative is available at www.strobe-statement.org.

For peer review only - http://bmjopen.bmj.com/site/about/guidelines.xhtml
